# Synthesis and Optoelectronic Characterization of Perylene Diimide-Quinoline Based Small Molecules

**DOI:** 10.3390/molecules24234406

**Published:** 2019-12-02

**Authors:** Stefania Aivali, Loukia Tsimpouki, Charalampos Anastasopoulos, Joannis K. Kallitsis

**Affiliations:** 1 Department of Chemistry, University of Patras, University Campus, Rio-Patras GR26504, Greece; aivali.s@upnet.gr (S.A.); loukia789@gmail.com (L.T.); xanastasop@upatras.gr (C.A.); 2 Foundation for Research and Technology Hellas/Institute of Chemical Engineering Sciences (FORTH/ICE-HT), Platani Str., Patras GR26504, Greece

**Keywords:** functional perylene, Suzuki reaction, non fullerene acceptors, electrochemical and optical properties

## Abstract

Perylene diimide (PDI) is one of the most studied functional dyes due to their structural versatility and fine tuning of the materials properties. Core substituted PDIs are prominent n-type semiconductor materials that could be used as non-fullerene acceptors in organic photovoltaics. Herein, we develop versatile organic building blocks based on PDI by decorating the PDI core with quinoline groups. Styryl and hydroxy phenyl mono and difunctionalized molecules were prepared using mono-nitro and dibromo bay substituted PDIs by Suzuki coupling with the respective boronic acid derivatives. A novel methodology using nitro-PDI under Suzuki coupling conditions as an electrophile partner was successfully tested. Furthermore, the PDI derivatives were used for the synthesis of soluble, electron accepting small molecules combining PDI with weak electron withdrawing quinoline derivatives. The new molecules presented wide absorbance in the visible spectrum from 450 to almost 700 nm while their LUMO levels and their energy levels are in the range of −3.8 to −4.2 eV.

## 1. Introduction

Perylene diimide (PDI) is known as promising *n*-type semiconductor building block and has attracted research interest owing to its unique versatile molecular structure, thermal stability, excellent electron affinity and outstanding optical properties [1,2,3,4]. These advantages have opened the route for various applications, like fluorescent emitters [5], organic field effect transistors (OFET) [6,7] MOS transistors [8] and organic photovoltaics OPVs [9,10,11]. Its structure allows easy functionalization at different positions (ortho, bay or imide) providing PDIs with significantly different optical, electronic and morphological properties [12,13]. In the field of organic photovoltaics, many efforts focused on the development of more efficient electron-donors or electron-acceptors, the control and stabilization of their morphology and new device architectures have provided power conversion efficiency (PCE) up to 16% [14,15]. Concerning the electron acceptors, non-fullerene acceptors (NFAs) have shown a dramatic increase on the OPVs efficiency [10,11,16]. Among them, PDI is one of the most promising candidate providing high electron mobility, good electron accepting character and low energy levels to afford PCEs over 10% [17,18]. Substitution with different groups at various positions in the PDI twist the planar core and suppress the strong intermolecular π–π stacking between perylene cores causing aggregates and resulting in decrease of the PCE in OPVs [9,19,20,21,22,23]. Depending on the position of functionalization, the PDI based NFAs can be categorized into bay, ortho and imide functionalized derivatives. Imide substitution enhances the solubility but has a small effect on the optical and energy characteristics, affording low efficiencies [24]. Bay substitution with various groups (donor or acceptor type) has a stronger effect on the optical and electronic properties improving also the solubility of the PDI, giving the possibility to tune the dye’s properties [25,26]. Phenyl substitution at the bay area has been used as a tool to disrupt the aggregation tendency [20,27]. Additionally, two or more PDIs in the same molecule have been studied, affording non-planar PDI-based dimers or more complicated 3D structures. Combining other semiconducting electron accepting or electron donating moieties is an efficient route towards controlling the optical and energy levels of the final molecules [28,29,30].

In this work, substitution at the bay area of the PDI was studied through Suzuki coupling. For the synthesis of difunctional PDIs, dibromo PDI was used. For the substitution at one site of the perylene skeleton, a nitro group selectively was added in a single position and used as an alternative electrophilic coupling partner for the Suzuki reaction, a novel strategy recently reported in the literature [31]. Hydroxy phenyl and styryl groups were selected to be attached to the perylene core, creating two different monofunctional and difunctional PDIs. Then, they were used for the synthesis of molecules combining PDI with weak electron accepting moieties based on quinoline. Generally, quinolines were shown as electron-transport molecules and have been studied by our group combined with fullerene derivatives, as electron acceptor materials or additives for OPVs [32,33,34]. Herein, 6-bromo-(2-pyridinyl)-4-phenyl-quinoline (Br-QPy) [35,36,37] or 6-bromo-(2-perfluorophenyl)-4-phenyl-quinoline (Br-5FQ) [32] and 6-phenyl-(2-perfluorophenyl)-4-phenyl-quinoline (Ph5FQ) were used as substituents at the bay area of PDI, using two different synthetic strategies to afford non-planar PDI molecules. The final molecules showed high solubility and were evaluated in terms of their optical and electrochemical characteristics and all share the same advantages imposed by the PDI units, forming non-planar structures of PDI with redshifted absorption profiles and low lying LUMOs.

## 2. Results and Discussion

### 2.1. Synthesis of Mono and Difunctional Perylene Diimide Derivatives

**Scheme 1:**
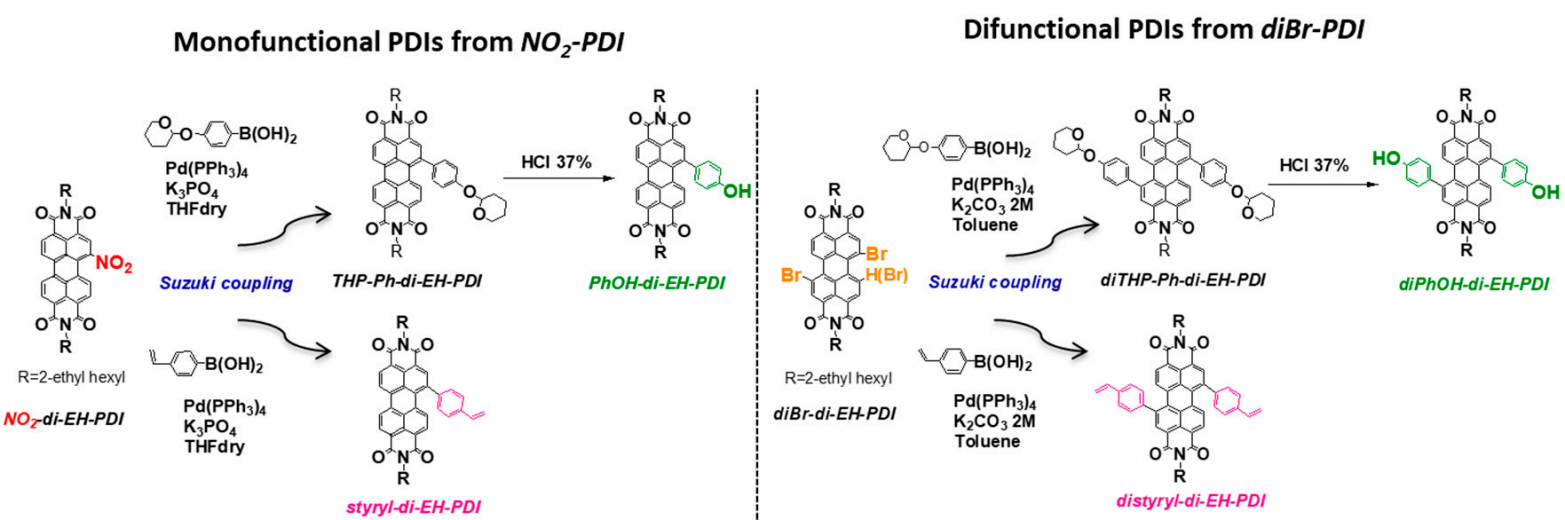
Synthetic procedure for monofunctional and difunctional Perylene diimides (PDIs).

Bay-substituted PDIs could be synthesized reacting mono- or di-bromo derivatives of PDI under transition-metal-catalyzed C−C Suzuki coupling conditions [11,12,38] However, there are several challenges. The bromination of the perylene skeleton under controlled conditions affords a 1,7- and 1,6-dibromo regioisomeric mixture, that is arduous to separate. Regarding the mono-bromo derivative of perylene diimide, can be achieved under mild conditions but in low yield and after a tedious purification process [39]. The synthetic routes providing the new monofunctional and difunctional PDIs are presented in Scheme 1. Firstly, the branched 2-ethylhexyl alkyl chains have been introduced to the starting commercially available material perylene tetracarboxylic dianhydride (PTCDA) through a condensation reaction, according to a literature reported procedure [40]. The final *N*,*N*′-Bis (2-ethylhexyl)-3,4,9,10-perylenetetracarboxylic diimide (di-EH-PDI) provide sufficiently soluble PDIs that could be used for the next step. For the difunctional PDIs, a bromination reaction was performed to have reactive groups in the bay area of the perylene skeleton. The two regioisomers arising from the bromination (1,6 and 1,7-di brominated PDI) could not been separated and used as a mixture according to literature (diBr-di-EH-PDI) [39]. The diBr-di-EH-PDI reacted through a Suzuki cross-coupling reaction with 4-styryl boronic acid group, providing two styryl groups attached at the bay area of perylene (distyryl-di-EH-PDI). Also, diBr-di-EH-PDI reacted with the same methodology with 4-(2-tetrahydropyranyloxy) phenylboronic acid [41] to give the diTHP-Ph-di-EH-PDI derivative, which with treatment with HCl in tetrahydrofouran (THF) solution, resulted to the desired diPhOH-di-EH-PDI. Regarding the monofunctional derivatives, in order to avoid tedious process for the mono bromo PDI isolation [39], selective addition of the nitro group to a single site of the perylene skeleton through controlled nitration conditions was utilized (at room temperature and in short time), enabling the isolation of the mono derivative in a multigram scale and in a very good yield (90%) without using column chromatography [42]. Mono nitro PDI derivative (NO_2_-di-EH-PDI) was used in palladium catalyzed Suzuki reaction, where the nitro group acts as the leaving group instead of another halogen. To the best of our knowledge mononitrated PDI has been used in Suzuki coupling reactions only one time before [43]. So, in order to investigate the advantage of the selective addition of the nitro group, we proceeded with the reaction of the nitro PDI with 4-styryl boronic acid and 4-(2-tetrahydropyranyloxy) phenylboronic acid in the presence of Pd(PPh_3_)_4_, potassium phosphate (K_3_PO_4_) and distilled tetrahydrofuran (THF dry) to synthesize styryl-di-EH-PDI and PhOH-di-EH-PDI (Scheme 1) in a fairly good yield (~80%). The final products presented good solubility in common organic solvents such as toluene, THF, dichloromethane (DCM), chloroform (CHCl_3_) and *ortho*-dichlorobenzene (*o*-DCB) and characterized through ^1^H NMR and ^13^C NMR spectroscopy (Figures S1, S2, S12 and S13).

**Figure 1:**
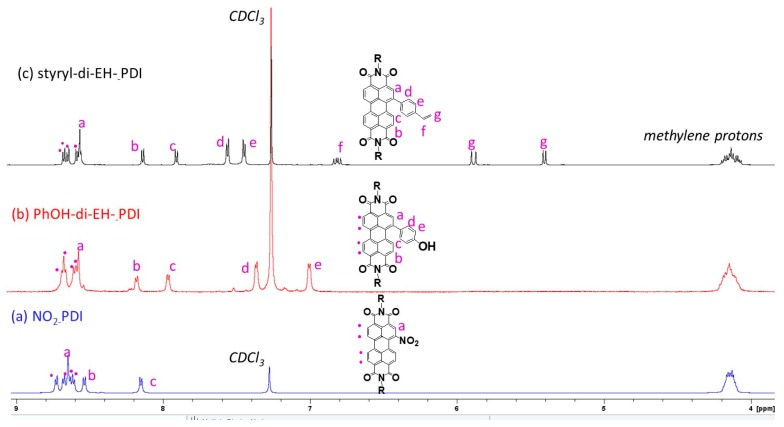
ΝΜR spectra of the expanded region 4.0–9.0 ppm of (**a**) NO_2_-di-EH-PDI, (**b**) PhOH-di-EH-PDI, and (**c**) styryl-di-EH-PDI in CDCl_3._

Representatively, ^1^H NMR spectra (expanded area from 4.00–9.00 ppm) of NO_2_-di-EH-PDI and of the monofunctional PDIs are presented in Figure 1. Regarding the NO_2_-di-EH-PDI successful synthesis was confirmed through the ^1^H NMR spectrum where the seven aromatic protons of perylene skeleton are observed together with the aliphatic protons of the ethylhexyl chain in agreement with literature [42]. More specifically, in the aromatic region, between 8.6 and 9.00 ppm 5 protons, (4 doubles and 1 singlet (a) overlapped), could be assigned and together with the two doublets at 8.5–8.6 ppm and 8.1–8.2 ppm we assign the seven protons of the aromatic backbone. With the introduction of the two functional groups at the perylene skeleton, the aromatic peaks from 8.6–9.0 ppm do not have a significant change, whereas b and c protons are shifted upfield due to electron donating character of these units. In the ^1^H NMR spectrum of PhOH-di-EH-PDI in CDCl_3_, (Figure 1b) characteristic peaks of PDI are present. Due to the hydroxyl group and the formation of aggregates via hydrogen bonding, broad peaks are observed in this spectrum. At 4.1 ppm, peak owing to the ethylhexyl methylene protons of the imide moiety and at the aromatic region, between 8.0 and 9.0 ppm, peaks of the seven aromatic protons of PDI skeleton could be assigned. The perspective peaks of the phenyl hydroxy group are presented at 7.0–7.5 ppm (d and e). In the ^1^H NMR spectrum of styryl-di-EH-PDI in CDCl_3_, (Figure 1c), apart from the characteristic peaks of PDI, peaks attributed to the styryl moiety between 7.40 and 7.60 ppm (aromatic protons of phenyl group) and at 5.5–6.5 ppm ascribed to the protons of double bond are also observed. The successful introduction of functional groups at two positions of the perylene skeleton is also observed in the ^1^H NMR spectra of distyryl-di-EH-PDI and diPhOH-di-EH-PDI (See Figures S3 and S4).

### 2.2. Synthesis of Quinoline Bay-Substituted Perylene Diimide Derivatives

After the synthesis and the characterization of the above PDIs, they were used for the synthesis of molecules combining perylene diimide linked with weaker electron accepting quinoline-based molecules. Quinolines are consider to be molecules with electron acceptor character, providing also good electron transport characteristics that could be beneficial. In previous studies, quinoline derivatives have been combined with fullerene derivatives to afford hybrid materials with combined optoelectronic properties and increased solubility. Herein, we combined the quinoline molecules with a non-fullerene acceptor, perylene diimide, in order to evaluate the effect on its optical and electronic properties.

Firstly, perfluorophenyl quinoline derivative was selected due to its extra electron withdrawing pentaflurophenyl group that can maintain the LUMO levels low lying and afford good solubility and stability properties to the final products. Our attempt was to react 6-bromo-(2-perfluorophenyl)-4-phenyl-quinoline (Br-5FQ) with styryl-di-EH-PDI and distyryl-di-EH-PDI, through a C-C Heck coupling in dimethylformamide (DMF) utilizing triethylamine as the proton scavenger and Pd(OAc)_2_ as the catalyst to compose 5FQ-styryl-di-EH-PDI and di5FQ-styryl-di-EH-PDI. The styryl group connecting the two moieties is a conjugated “bridge” that provides fully electronic interaction between two molecules and increases conjugation. A key step in this palladium-catalyzed process is alkene insertion into the Pd−C bond. This reaction seemed to be challenging because perfluroaryls M−Rf σ-bonds between transition metals and perfluorinated groups are particularly strong and reluctant to reaction [44]. The final products were isolated after column chromatography in very low yields up to 30%. The ^1^H NMR spectrum of the final 5FQ-styryl-di-EH-PDI, (Figures S5 and S6) and di5FQ-styryl-di-EH-PDI (Figure S7), the absence of characteristic peaks at 5.5–6.5 ppm that could be attributed to double bonds combined with characteristic peaks of 2-ethylhexyl chain aliphatic protons and aromatic protons of quinoline and PDI that can be observed between 7.0 and 8.8 ppm confirm the successful synthesis. In Figure S6, the aromatic region of the ^1^H NMR spectrum of the 5FQ-styryl-di-EH-PDI is presented, together with the ^1^H NMR spectra of the Br-5FQ and styryl-di-EH-PDI precursors. Although in the final product all the characteristics protons of the moieties could be assigned, peaks attributed to side products of the Heck reaction that could not be removed after column chromatography purification are observed.

**Scheme 2:**
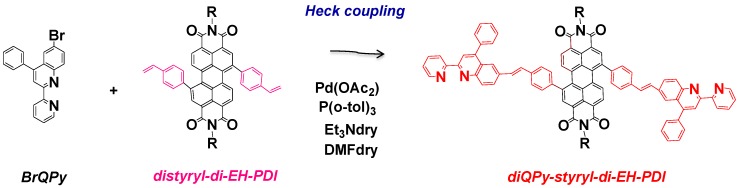
Synthetic procedure for the synthesis of diQPy-styryl-di-EH-PDI.

Due to the above problems, 6-bromo-(2-pyridinyl)-4-phenyl-quinoline (Br-QPy) was chosen to react with the distyryl-di-EH-PDI under the same Heck coupling conditions utilizing triethylamine as base and Pd (OAc)_2_ as the catalyst in DMF solution as shown in Scheme 2. Quinoline pyridine derivative consider to possess electron acceptor character and has been used previously in donor–acceptor copolymers for non-covalent interactions with fullerene species [34]. Herein, was used for the synthesis of non-planar, PDI structures affording molecules with low lying LUMOs, increased conjugation and good solubility.

**Figure 2:**
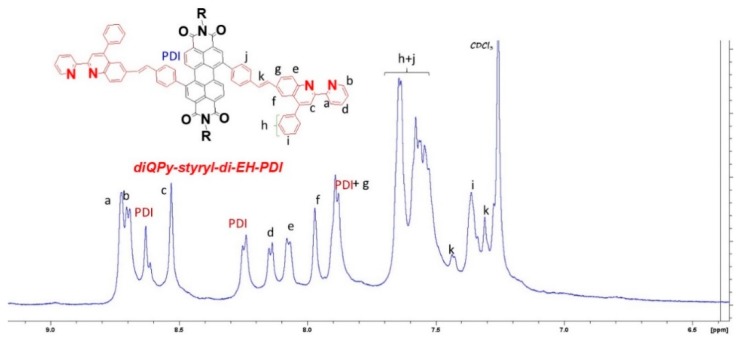
^1^H ΝΜR spectra in the zoomed region 7.0–9.0 ppm of (**a**) PhOH-di-EH-PDI and (**b**) Ph5FQ-PhO-di-EH-PDI in CDCl_3._

The final molecule named diQPy-styryl-di-EH-PDI afforded in yields up to 60% after recrystallization from toluene. Through ^1^H and ^13^C NMR characterization (Figures S8 and S15, respectively) the successful synthesis was confirmed. In Figure 2, expanded aromatic region of the ^1^H NMR spectrum of the diQPy-styryl-di-EH-PDI is presented where all the characteristic peaks of the aromatic protons of quinoline-pyridine and PDI could be assigned confirming the successful synthesis.

**Scheme 3:**
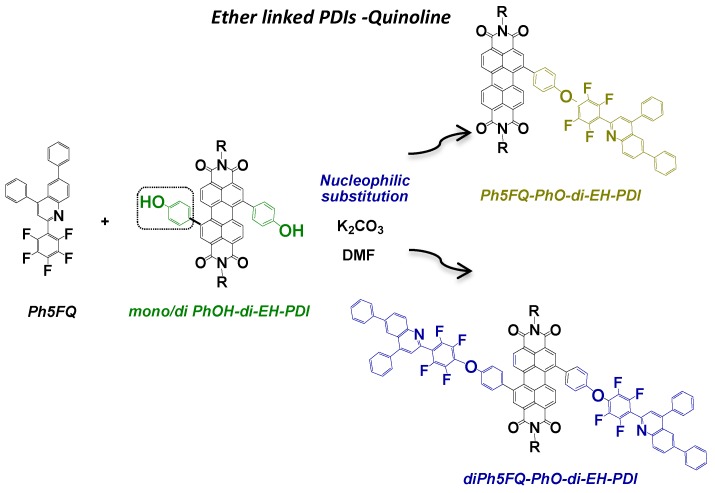
Synthetic procedure for the synthesis of ether-linked PDIs–Quinoline molecules.

In the next step, in order to connect the PDI with the perfluorophenyl quinoline group a facile, metal-free way was investigated. Taking advantage of the highly reactive perfluorophenyl group under basic conditions [45], PhOH-di-EH-PDI and diPhOH-di-EH-PDI molecules reacted with the “para” fluorine atom of phenyl perfluorophenyl quinoline under typical basic conditions of nucleophilic substitution as shown in Scheme 3. Br-5FQ was replaced with the phenyl perfluorophenyl quinoline moiety (Ph5FQ) in order to have a single reactive site. The isolation of ether based Ph5FQ-PhO-di-EH-PDI and diPh5FQ-PhO-di-EH-PDI molecules was achieved after purification with column chromatography in good yields up to 80%.

**Figure 3:**
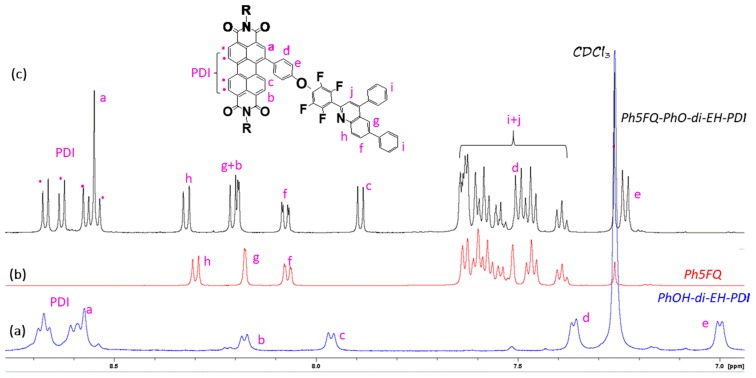
^1^H ΝΜR spectra in the zoomed region 7.0–9.0 ppm of (**a**) PhOH-di-EH-PDI, (**b**) Ph5FQ and (c) Ph5FQ-PhO-di-EH-PDI in CDCl_3__._

The final molecules were identified through ^1^H NMR and ^13^C spectroscopy (Figures S9, S10, S16 and S17, respectively). Representatively, in Figure 3, enlarged region of the ^1^H NMR spectrum of the final Ph5FQ-PhO-di-EH-PDI is presented. Comparing the latter with the ^1^H NMR spectra of the two precursors (PhOH-di-EH-PDI and Ph5FQ), characteristic peaks of the aromatic protons of quinoline and PDI can be assigned and confirm the successful synthesis.

**Figure 4:**
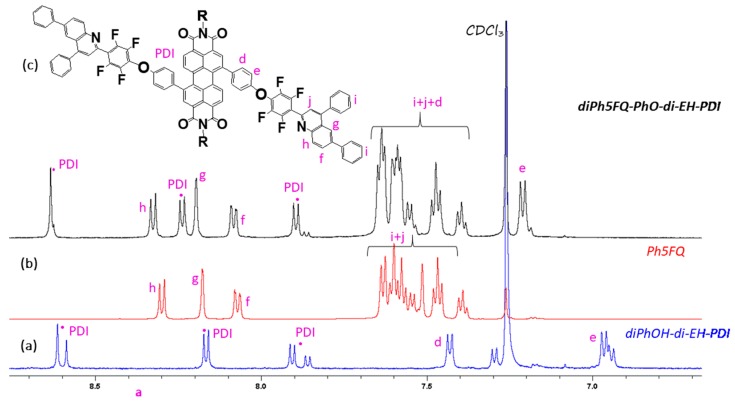
^1^H ΝΜR spectra in the zoomed region 7.0–9.0 ppm of (**a**) diPhOH-di-EH-PDI, (**b**) Ph5FQ and (**c**) diPh5FQ-PhO-di-EH-PDI in CDCl_3_.

In the ^1^H NMR spectra of diPh5FQ-PhO-di-EH-PDI (Figure 4), characteristic peaks of the aromatic protons of quinoline and PDI can also be observed confirming the successful synthesis.

**Figure 5:**
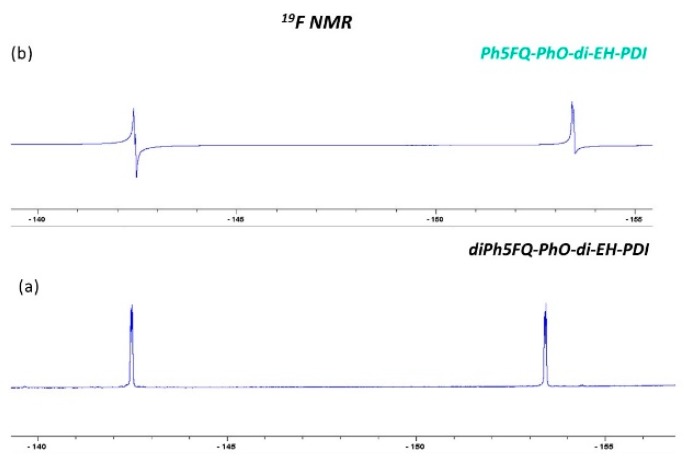
^19^F ΝΜR spectra of (**a**) diPh5FQ-PhO-di-EH-PDI and (**b**) Ph5FQ-PhO-di-EH-PDI in CDCl_3_.

^19^F NMR was investigated in order to confirm the successful substitution of the fluorine at the *para* position. In Figure 5a,b, ^19^F NMR spectra of diPh5FQ-PhO-di-EH-PDI and Ph5FQ-PhO-di-EH-PDI are presented respectively, where the two types of fluorine’s could be observed and the peak owing to the fluorine at the *para* position was disappeared, confirming the successful nucleophilic substitution.

The ATR-IR spectra of the mono substituted and disubstituted PDIs are presented in Figure S11. Characteristic absorption bands of PDI are observed (1695 and 1655 cm^−1^ (C=O imide), (2955 and 2924 cm^−1^ C-H stretching of aliphatic chains), (1590 and 1505 cm^−1^ aromatic). For the PhOH-di-EH-PDI and diPhOH-di-EH-PDI a broad absorption band at 3350 cm^−1^ due to O-H stretching can also observed confirming the successful introduction of the phenol to PDI. For the mono and di Ph5FQ-PhO-di-EH-PDI an additional absorption band attributed to the ether bond at 1210 cm^−1^ is also observed. The solubility of mono substituted and disubstituted PDIs was investigated in common organic solvents and the results are presented in Table 1 and Table 2 respectively. Good solubility for all functional molecules could be observed. The di-Ph5FQ-PhO-di-EH-PDI is soluble in all the solvents we tested, whereas the Ph5FQ-PhO-di-EH-PDI is soluble only in chloroform and *o*-DCB solutions. The diQPy-styryl-di-EH-PDI is soluble in all tested solvents.

### 2.3. Optical and Electrochemical Properties

**Figure 6:**
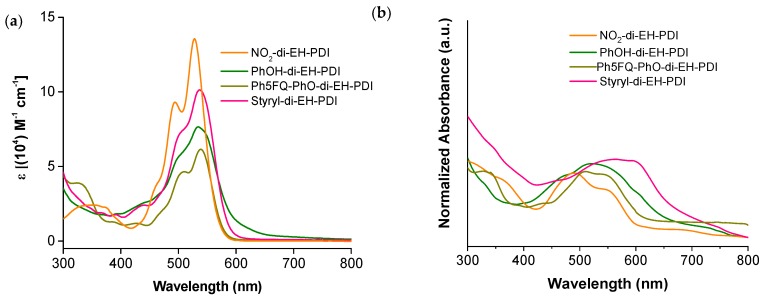
UV-Visible absorption of NO_2_—di-EH-PDI, PhOH-di-EH-PDI, Ph5FQ-PhO-di-EH-PDI and Styryl-di-EH-PDI (**a**) in *o*-DCB solutions (10^−5^ M) and (**b**) in film form.

**Figure 7:**
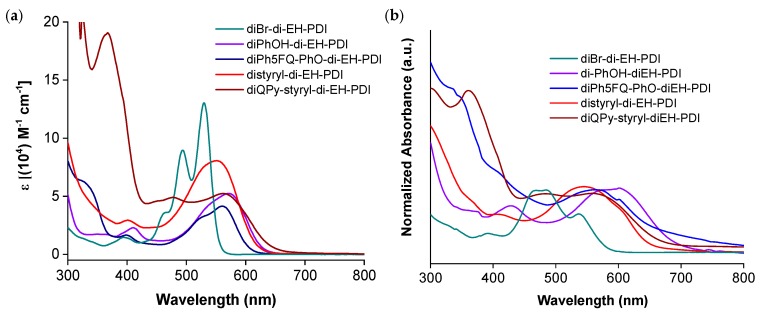
UV-Visible absorption of diBr-di-EH-PDI, diPhOH-di-EH-PDI, diPh5FQ-PhO-di-EH-PDI distyryl-di-EH-PDI and diQPy-styryl-di-EH-PDI (**a**) in *o*-DCB solutions (10^−5^ M) and (**b**) in film form.

The substitution of PDI in the bay area is known to distort the planar aromatic core affecting the self-assembly of the molecule together with its optical and electronic properties. The absorbance and electronic properties of all the new synthesized small molecules are shown in Table 3. The ultraviolet -visible (UV-Vis) absorption properties of all the molecules synthesized above were investigated in solution (10^−5^ M) and in film form. Figure 6a shows the UV-visible absorption spectra of mono substituted PDIs (NO_2_ –di-EH-PDI, styryl-di-EH-PDI, PhOH-di-EH-PDI, and Ph5FQ-PhO-diEHPDI) in *o*-DCB solutions (10^−5^ M) and Figure 6b in film form. gi shows the UV-visible absorption spectra of di substituted PDIs (diBr –di-EH- PDI, distyryl-di-EH-PDI, diPhOH-di-EH-PDI, diQPy-styryl-di-EH-PDI and diPh5FQ-PhO-diEHPDI) in *o*-DCB solutions (10^−5^ M) and Figure 7b in film form.

Generally, substitution with electron donating groups at the bay area of the PDI causes a redshift in the absorption due to electronic interactions between perylene skeleton and electron donating moieties [12]. In the UV-Vis spectra of mono functional PDIs, styryl-di-EH-PDI and PhOH-di-EH-PDI, a bathochromic shifted absorption band (λmax = 540 nm) relative to the NO_2_-di-EH-PDI could be observed. For the difunctional molecules, distyryl-di-EH-PDI and diPhOH di-EH-PDI, a larger bathochromic shifted absorption band (λ_max_ = 556 and 570 nm respectively) is observed, due to stronger intermolecular interactions and more twisted perylene core. Also, a second absorption band exists around 400 nm that can be attributed to the transition from the ground state to a higher excited state [10]. In film form, monofunctional PDIs show a broader absorption profile with a maximum absorption peak at 570 nm for the styryl-di-EH-PDI and at 540 nm for the PhOH-di-EH-PDI molecule. For the difunctional PDIs, diPhOH-di-EH-PDI presents a clear red shifted of 25 nm (λ_max_ = 595 nm) compared to the absorbance spectrum in the solution, indicating the creation of aggregates in the film form probably due to non-covalent interactions of hydroxy groups. Notably, distyryl-di-EH-PDI does not present red shifted absorbance in film form, indicating suppression of aggregation probably due to torsion between the perylene core and the double bond.

The substitution with quinoline groups was also investigated in terms of their optical properties. Ether based molecules containing one or two quinoline moieties (Ph5FQ-PhO-di-EH-PDI and diPh5FQ-PhO-di-EH-PDI) present the same characteristics with their initial phenol functionalized PDI, having an extra absorbance band at 330 nm due to the perfluorophenyl quinoline group. On the other hand, styryl linked diQPy-styryl-di-EH-DI presented a lower absorbance band at the area of PDI’s absorbance and an intense absorption band around 365 nm caused from the quinoline moieties. The C-C coupling provides an all conjugated path that more significantly affects the optical properties of the two groups, whereas the oxygen atom interrupts the conjugation isolating PDI and quinoline moieties.

The electrochemical properties of all synthesized PDI derivatives were also investigated. The highest occupied molecular orbital (HOMO) and lowest unoccupied molecular orbital (LUMO) energy levels were calculated by combining both absorption spectroscopy and cyclic voltammetry (CV). The LUMO energy levels were calculated from the first reduction onset potential (E_RED_) using as reported equation giving in Experimental Part. The HOMO energy levels were calculated from LUMO levels and the optical bandgap due to strong *n*-type characteristics of the synthesized acceptors from the equation E_opt_ = HOMO-LUMO.

The E_opt_ (optical bandgap) of the molecules was determined from the equation [46]: $E_{\mathrm{opt}}=\frac{1240}{\lambda_{\mathrm{onset}}}$. λ_onset_ was determined by the lowest energy onset wavelength of the absorbance in film form. The optical band gaps were found between 1.69 and 1.96 eV.

**Figure 8:**
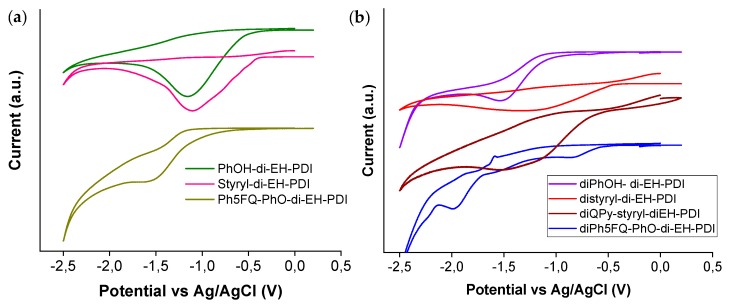
Reductive cyclic voltammograms spectra of (**a**) monosubstituted PDIs and (**b**) disubstituted PDIs.

Figure 8a presents the typical reductive voltammograms of monosubstituted PDIs and Figure 8b of disubstituted molecules. The synthesized PDI derivatives presented good electron accepting properties with LUMO levels in the range of −3.8 V to −4.2 eV. This is in a good agreement with typical PDI molecules. The LUMO energy levels of the widely used fullerenes such as PCBM are known to be around −4.0 eV so these molecules could be appropriate candidates to act as electron accepting materials with a variety of electron donors, matching carefully their energy levels in order to find the most efficient combination. The monofunctional and difunctional PDIs, mono/di styryl-di-EH-PDI and mono/di PhOH-di-EH-PDI provide low lying LUMO levels, lower than −4.0 eV, highlighting that could be used as new building blocks for the synthesis of new small molecules based on PDI. Additionally, the PDI molecule containing the quinoline pyridine group maintains its good electron accepting character with estimated LUMO at −3.95 eV. We could conclude that the electron affinities of the final materials are dictated by the PDI units resulting in low lying LUMOs.

**Figure 9:**
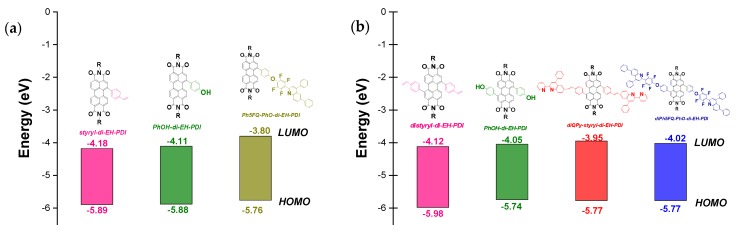
Energy levels of (**a**) monosubstituted PDIs and (**b**) disubstituted PDIs.

In Figure 9, the energy level diagram of monosubstituted PDIs (Figure 9a) and of disubstituted PDIs (Figure 9b) are provided to sum up their electronic properties.

## 3. Materials and Methods

### 3.1. Materials

Perylene-3,4,9,10-tetracarboxylic dianhydride 97% (PTCDA), 2-ethyl-1-hexylamine 98%, bromine ≥99.99%), fuming nitric acid >90%, 4-styryl boronic acid ≥95%, phenyl boronic acid ≥97%, magnesium sulphate anhydrous >99.5% (MgSO_4_), sodium sulphite >98% Na_2_SO_3_ were purchased from Merck (Darmstadt, 64293, Germany). Tetrahydrofuran was purchased also from Merck and was freshly distilled with benzophenone and metallic sodium (THF (dry)). All other solvents and reagents were purchased from Aldrich or Alfa Aesar and were used without further purification unless otherwise stated. 4-(2-tetrahydropyranyloxy) phenylboronic acid [41], 6-Bromo phenyl-(2-perfluorophenyl)-4-phenyl-quinoline (Br5FQ) [32], 6-phenyl-(2-perfluorophenyl)-4-phenyl-quinoline (Ph-5FQ) [33] 6-bromo-(2-pyridinyl)-4-phenyl-quinoline (Br-QPy) [35] and the catalyst palladium (II) tetrakis triphenyl2phosphine [Pd (PPh_3_)_4_] [47] were synthesized according to published procedures.

### 3.2. Methods

^1^H, ^13^C, ^19^F Nuclear Magnetic Resonance (NMR) spectra were recorded on a Bruker Advance (Bruker BioSpin GmbH, Magnet Division, Karlsruhe, Germany) DPX 600, 150, and 564 MHz spectrometer, respectively, with CDCl_3_ as solvent containing TMS as internal standard. Melting points recorded in Fisherbrand Digital Melting Point Apparatus. Microanalyses were performed on a Carlo Erba EA 1108 CHNS elemental analyzer. MALDI-TOF mass spectra were recorded on a Bruker Daltonik GmbH Autoflex Speed Maldi TOF/TOF Mass Spectrometer using α-cyano-4-hydroxycinnamic acid as the matrix dissolved in AcN/H2O/TFA. One microliter of the sample dilute solution of 1 µg/mL in dichloromethane was mixed with 1 uL of the matrix solution and spotted on a Bruker MTP 384 ground steel plate target. The spectra were analyzed using the flexAnalysis software.

Attenuated Total Reflectance (ATR) spectra were recorded on a “Bruker Optics’ Alpha-P Diamond ATR Spectrometer of Bruker Optics GmbH” (Ettlingen, Germany).

UV-Vis spectra were recorded using a Hitachi U-1800 spectrophotometer (Hitachi High-Technologies Europe GmbH, Mannheim, Germany). All UV-Vis measurements were performed in air using quartz cuvettes and flat quartz substrates for the examination of solutions and films, respectively.

The electrochemical behavior of the fabricated materials was investigated using cyclic voltammetry (CV). CV experiments were carried out in a three electrode cell [48]. Two kinds of working electrodes have been used. An ITO/Glass with the synthesized sensitizers to be measured drop-casted on the ITO conductive side and a dye-sensitized TiO_2_/FTO/Glass. An Ag/AgCl electrode served as reference and a platinum wire was used as the counter electrode. Thin films of the fabricated materials were drop casted on ITO coated glass slides, (R_sheet_ < 15 Ω/square), preheated at 80 °C for 20 min, from precursor solutions of chloroform. The resulting films were further annealed at 80 °C for 15 min. An Autolab PGSTAT 302 N electrochemical analyzer connected to a personal computer running the NOVA 1.8 software was used for data collection and analysis. All experiments were carried out at a scan rate of 0.1 V/s. Tetrabutylammonium hexafluorophoshate (TBAPF_6_) 0.1 M in Acetonitrile (CH_3_CN) was used as supporting electrolyte. Before carrying out the measurements the cell was purged with pure argon for 20 min to remove diluted gasses. The reference electrode potential was calibrated against Ferrocene/Ferrocenium (Fc/Fc+) after each voltammetry run.

The LUMO energy levels were calculated from the first reduction onset potential using as reported the equation [48]:

E_LUMO_ = e (E_REDonset_ − E_1/2Ferrocene_) − 5.2 [eV] (1)

E_REDonset_ = the onset determined for the reduction peak of each molecule in cyclic voltammetry (V) versus Ag/AgCl.

E_1/2Ferrocene_ = (E_red_ + E_ox_)/2 vs. Ag/AgCl.

### 3.3. Synthesis of Functional Perylene Diimide Molecules and Perylene-Quinoline Based Molecules.

#### 3.3.1. Synthesis of Styryl-di-EH-PDI

A 250 mL round bottom flask, equipped with a reflux condenser and a magnetic stirrer, was degassed (flamed under vacuum) and filled with argon. One gram (1.52 mmol) NO_2_-di-EH-PDI, 0.247 g (1.67 mmol) 4-styryl boronic acid, 1.13 g (5.31 mmol) K_3_PO_4_, 0.227 g (0.197 mmol) Pd(PPh_3_)_4_ and 120 mL tetrahydrofuran dry were added. The system was degassed, flushed with argon again and was heated to reflux temperature for 1 day. The reaction mixture was allowed to cool to room temperature then the solution was filtered from celite and the solvent was rotary evaporated. After evaporation of the solvent, the mixture was precipitated into methanol in MeOH for further purification, was filtered and washed with distilled water, hexane and MeOH. The obtained solid was dried under vacuum at 40 °C overnight. Yield: 0.8693 g (80%). m.p. 198–200 °C. ^1^H NMR (600 MHz, CDCl_3_): δ (ppm) = 8.68–8.64 (d, 1H) 8.64–8.60 (d, 1H) 8.59–8.50 (3H, m) 8.15–8.10 (d, 1H) 7.92–7.87 (d, 1H) 7.59–7.53 (d, 1H) 7.47–7.41 (d, 2H) 6.85–6.76 (q, 1H) 5.93–5.82 (d, 1H) 5.45–5.30 (d, 1H) 4.22–4.03 (m, 4H), 2.02–1.89 (m, 2H) 1.26–1.45 (m, 16H), 0.97–0.85 (m, 12H); ^13^C NMR (150 MHz, CDCl_3_): δ (ppm) = 163.8, 163.67, 163.64, 141.7,141.4, 137.9, 135.9,134.8, 134.7, 134.4, 132.5, 131.0, 130.8, 130.1, 130.0, 128.8, 128.7, 128.2, 128.1, 128.0, 127.4, 123.5, 123.2, 123.0, 122.6, 122.2, 115.3, 44.3, 44.29, 38.0, 37.9, 30.8, 30.79, 28.7, 28.6, 24.1, 24.0, 23.0, 14.1, 10.63, 10.61 MS (MALDI-TOF) Calcd. for C_48_H_48_N_2_O_4_: 716,36 found: [M + H] 717.53.

#### 3.3.2. Synthesis of PhOH-di-EH-PDI

A 250 mL round bottom flask, equipped with a reflux condenser and a magnetic stirrer, was degassed (flamed under vacuum) and filled with argon. Next, 0.700 g (1.061 mmol) NO_2_-di-EH-PDI, 0.283 mg (1.27 mmol) 4-(2-tetrahydropyranyloxy) phenyl boronic acid, 788 mg (3.72 mmol) K_3_PO_4_, 159 mg (0.138 mmol) Pd (PPh_3_)_4_ and 84 mL tetrahydrofuran dry were added. The system was degassed, flushed with argon again and was heated to reflux temperature for 1 day. The reaction mixture was allowed to cool to room temperature then the solution was filtered from celite and the solvent was rotary evaporated. After evaporation of the solvent, the mixture was precipitated into methanol in MeOH for further purification, was filtered, washed with distilled water, and dried under vacuum at 40 °C overnight. The obtained solid was added in a 50 mL bottom flask with a reflux condenser, a magnetic stirrer and 20 mL tetrahydrofuran. Afterwards, 2 mL HCl 37% were added and the system was heated to reflux temperature for 3 hours. The solution was cooled, was poured in MeOH, filtered and washed with H_2_O, hexane and MeOH. The obtained solid was recrystallized from toluene and dried under vacuum at 40 °C overnight. Yield: 0.5625 g (75%). m.p. 280–282 °C. ^1^H NMR (600 MHz, CDCl_3_): δ (ppm) = 8.70–8.53 (m, 5H), 8.20–8.151 (d, 1H), 8.00–7.93 (d, 1H), 7.40–7.31 (d, 2H), 7.05–6.95 (d, 2H), 4.22–4.03 (m, 4H), 2.02–1.89 (m, 2H) 1.26–1.45 (m, 16H), 0.97–0.85 (m, 12H); ^13^C NMR (150 MHz, CDCl_3_): δ (ppm) = 163.9, 163.8, 163.7, 156.3, 141.5, 136.3, 135.0, 134.8, 134.8, 134.6, 132.5, 131.1, 130.8, 130.2, 130.0, 129.7, 128.9, 128.2, 128.1, 127.6, 123.5, 123.2, 123.0, 122.6, 122.2, 122.1, 117.35, 44.3, 44.29, 38.0, 37.9, 30.8, 30.79, 28.7, 28.6, 24.1,24.0, 23.0, 14.1, 10.63, 10.61. Elemental analysis for C_46_H_46_N_2_O_5_ calcd. C 78.16, H 6.56, N 3.96; found C 78.02, H 6.49, N 3.95.

#### 3.3.3. Synthesis of diPhOH-di-EH-PDI

A 250 mL round bottom flask, equipped with a reflux condenser and a magnetic stirrer, was degassed (flamed under vacuum) and filled with argon. 2.90 g (3.75 mmol) diBr-di-EH-PDI, 2.00 g (9.009 mmol) 4-(2-tetrahydropyranyloxy) phenylboronic acid, 3.10 g (22.5 mmol) K_2_CO_3_ in 2 M aqueous solution, 0.173 g (0.150 mmol) Pd(PPh_3_)_4_ and 100 mL toluene were added. The system was degassed, flushed with argon again and heated to reflux temperature for 2 days. After, the solution was filtered from paper filter followed by extraction of the organic layer with toluene and distilled water. The organic part was stirred with magnesium sulfate (MgSO_4_), filtrated and the solvent was rotary evaporated. Then, the solid was dispersed in MeOH for further purification, filtered, washed with H_2_O and was dried under vacuum at 40 °C overnight. Then the solid was added in a 50 mL bottom flask with a reflux condenser, a magnetic stirrer and 20 mL tetrahydrofuran. 4 mL HCl 37% were added and the system was heated to reflux temperature for 3 hours. The solution was cooled, poured in MeOH, filtered and washed with H_2_O and hexane and MeOH. The obtained solid was dried under vacuum at 40 °C overnight. Yield: 1.799 g, (60%) m.p. 363–365 °C. ^1^H NMR (600 MHz, CDCl_3_): δ (ppm) = 8.63–8.54 (d, 2H), 8.18–8.14 (d, 2H), 7.93–7.83 (m, 2H), 7.44–7.28 (m, 4H), 7.00–6.9 (m, 4H), 4.2–4.0 (m, 4H) 2.02–1.89 (m, 2H) 1.26–1.45 (m, 16H), 0.97–0.85 (m, 12H) ^13^C NMR is unavailable due to low solubility of the molecule. MS (MALDI-TOF) Calcd. for C_52_H_50_N_2_O_6_: 798.37 found: [M + H] 799.42.

#### 3.3.4. Synthesis of distyryl-di-EH-PDI

A 250 mL round bottom flask, equipped with a reflux condenser and a magnetic stirrer, was degassed (flamed under vacuum) and filled with argon. 1.50 g (1.94 mmol) diBr-di-EH-PDI, 0.661 g (4.47 mmol) 4-styryl boronic acid, 1.61 g (11.7 mmol) K_2_CO_3_ in 2 M aqueous solution, 0.0672 g (0.0583 mmol) Pd(PPh_3_)_4_ and 55 mL toluene were added. The system was degassed, flushed with argon again and was heated to reflux temperature for 2 days. After the solution was filtered from paper filter, followed by extraction of the organic layer with toluene and distilled water. The organic part was stirred with magnesium sulfate (MgSO_4_), filtrated and the solvent was rotary evaporated. The obtained solid was dried under vacuum at 40 °C overnight. Then the solid was dispersed in MeOH for further purification, was filtered and was washed with acetone. The solid was dried under vacuum at 40 °C overnight. Yield: 1.034 g (65%). m.p. >400 °C. ^1^H NMR (600 MHz, CDCl_3_): δ (ppm) = 8.60–8.59 (s, 1H) 8.59–8.58 (s, 1H) (8.16–8.09 (m, 2H) 7.87–7.81 (d, 2H), 7.69–7.64 (m,1H) 7.54–7.49 (m, 6H) 7.48–7.44 (m,1H), 6.85–6.76 (q, 1H) 5.93–5.82 (d, 1H) 5.45–5.30(d, 1H) 4.2–4.0 (m, 4H) 2.02–1.89 (m, 2H) 1.26–1.45 (m, 16H), 0.97–0.85 (m, 12H) ^13^C NMR (150 MHz, CDCl_3_) = 163.86, 163.83, 163.77, 149.2, 141.4, 140.7, 137.9, 135.9, 135.23, 134.89, 132.6, 130.5, 130.2, 129.5, 129.3, 129.1, 127.9, 127.6, 122.3, 115.2, 44.3, 37.9, 30.8, 28.7, 24.05, 23.03, 14.05, 10.62. Elemental analysis for C_56_H_54_N_2_O_4_ calcd. C 82.12, H 6.65, N 3.42; found C 81.88, H 6.64 N 3.46.

#### 3.3.5. Synthesis of diQPy-styryl-di-EH-PDI

A 25 mL round bottom flask, equipped with a reflux condenser and a magnetic stirrer, was degassed (flamed under vacuum) and filled with argon. 50 mg (0.0610 mmol) distyryl-di-EH-PDI, 50,72 mg (0.1404 mmol) BrQPy, 0.14 mg (0.0006 mmol) Pd(OAc)_2_, 1.11 mg (0.0037 mmol) P(*o*-tol)_3_, 1 mL triethylamine dry and 7 mL dimethylformamide dry were added. The system was degassed, flushed with argon again and was heated at 120 °C for 3 days. After, the solution was cooled, poured in MeOH, filtered, and washed with acetone. The obtained solid was dried under vacuum at 40 °C overnight. Recrystallization with toluene was followed and the obtained solid was dried under vacuum at 40 °C overnight. Yield: 0.0505 g (60%). m.p. 242–245 °C. ^1^H NMR (600 MHz, CDCl_3_): δ (ppm) = 8.66–8.61 (s, 2H), 8.26–819 (d, 2H) 8.25–8.21(d, 2H), 8.18–8.08 (m, 3H), 8.03–7.95 (dd, 2H), 7.92–7.82 (m, 2H) 7.70–7.65 (m, 2H) 7.63–7.48 (m, 6H), 7.45–7.40 (m, 3H) 7.30–7.27 (m, 2H), 7.19–7.16 (m, 2H) 4.15–4.03 (m, 4H), 2.09–2.01 (m, 2H), 1.90–1.71 (m, 16H), 1.03–0.95 (m, 12H). ^13^C NMR (150 Hz, CDCl_3_) = 163.8, 163.7, 156.2, 155.45, 149.2, 149.1, 148.5, 141.3, 140.56, 138.32, 137.57, 136.9, 135.46, 135.15, 134.7, 132.5, 132.4, 130.7, 130.5, 129.7, 129.5, 129.2, 128.7, 128.4, 128.3, 127.7, 127.0, 126.7, 124.9, 124.0, 122.3, 121.9, 121.8, 119.8, 44.3, 37.9, 30.8, 28.7, 24.05, 23.03, 14.05, 10.62 MS (MALDI-TOF) Calcd. for C_96_H_78_N_6_O_4_: 1379.61 found: [M + H] 1379.54.

#### 3.3.6. Synthesis of Ph5FQ-PhO-di-EH-PDI

A 10 mL round bottom flask, equipped with a reflux condenser and a magnetic stirrer, was degassed (flamed under vacuum) and filled with argon. Fifty milligrams (0.0707 mmol) PhOH-di-EH-PDI, 34.8 mg (0.0777 mmol) Ph5FQ, 29.3 mg (0.2122 mmol) K_2_CO_3_, and 6 mL dimethylformamide dry were added. The system was degassed, flushed with argon again and was heated at 90 °C overnight. After the solution was cooled, poured in H_2_O, was filtered and washed with MeOH. The obtained solid was dried under vacuum at 40 °C overnight. Purification was achieved by column chromatography (CHCl_3_, silica gel). Yield 0.6418 g (80%). m.p. 296–298 °C. ^1^H NMR (600 MHz, CDCl_3_): δ (ppm) = 8.69–8.65 (d, 1H), 8.65–8.61 (d, 1H), 8.59–8.52 (m, 3H), 8.34–8.30 (d, 1H), 8.22–8.18 (m, 2H), 8.09–8.05 (dd, 1H), 7.90–7.86 (d, 1H), 7.65–7.61(m, 4H) 7.61–7.52 (m, 4H), 7.51–7.44 (m, 4H) 7.41–7.39 (m, 1H), 7.25–7.20 (d, 1H), 4.20–4.04 (m, 4H), 2.09–2.01 (m, 2H), 1.90–1.71 (m, 16H), 1.03–0.95 (m, 12H); ^19^F NMR (564 MHz, CDCl_3_): δ (ppm) = −153.4, −142.4. ^13^C NMR (150 MHz, CDCl_3_): δ (ppm) = 163.8, 163.6, 163.5, 157.4, 149.7, 148.1, 140.6, 140.3, 138.1, 137.4, 136.0, 134.8, 134.6, 134.3, 132.6, 131.0, 130.9, 130.6, 130.2, 129.9, 129.6, 129.0, 128.9, 128.8, 128.3, 128.0, 127.9, 127.5, 127.5, 126.3, 123.5, 123.5, 123.2, 122.7, 122.4, 122.2, 117.7, 44.3, 44.29, 38.0, 37.9, 30.8, 30.79, 28.7, 28.6, 24.1,24.0, 23.07, 23.05, 14.1, 10.63, 10.61 MS (MALDI-TOF) Calcd. for C_73_H_59_F_4_N_3_O_5_: 1333.44 found: [M + H] 1334.34.

#### 3.3.7. Synthesis of di5FQ-PhO-di-EH-PDI

A 25 mL round bottom flask, equipped with a reflux condenser and a magnetic stirrer, was degassed (flamed under vacuum) and filled with argon. One hundred milligrams (0.1252 mmol) diPhOH-di-EH-PDI, 123 mg (0.275 mmol) Ph5FQ, 103 mg (0.751 mmol) K_2_CO_3_, and 8 mL dimethylformamide dry were added. The system was degassed, flushed with argon again and heated at 90 °C overnight. After the solution was cooled, was poured in H_2_O, filtered and purification was achieved by column chromatography (CHCl_3_, silica gel). The obtained solid was dried under vacuum at 40 °C overnight. Yield: 0.1672g (80%). m.p. 238–240 °C. ^1^H NMR (600 MHz, CDCl_3_): δ (ppm) = 8.66–8.61 (s, 2H), 8.35–8.29 (d, 2H) 8.25–8.21 (d, 2H), 8.21–8.17 (s, 1H) 8.10–8.06 (dd, 2H), 7.91–7.87 (d, 2H) 7.65–7.61 (m, 8H) 7.61–7.55 (m, 8H), 7.57–7.53 (d, 2H) 7.5–7.44 (t, 4H), 7.41–7.39 (m, 2H) 7.23–7.18 (m, 4H) 4.15–4.03 (m, 4H), 2.09–2.01 (m, 2H), 1.90–1.71 (m, 16H), 1.03–0.95 (m, 12H); ^19^F NMR (CDCl_3_; 564 MHz): −153.4, −142.4. ^13^C NMR (CDCl_3_, 150 MHz): δ (ppm) = 163.8, 163.7, 157.5, 149.7, 148.2, 146.9, 146.0, 144.4, 142.6, 141.0, 140.6, 140.3, 139.9, 137.8, 137.4, 135.3, 134.8, 132.6, 130.8, 130.6, 130.2, 129.9, 129.6, 129.3, 129.0, 128.9, 127.9, 127.8, 127.5, 126.3, 123.5, 123.4, 122.4, 122.2, 117.5, 44.3, 38.0, 30.8, 28.6, 24.0, 23.05, 14.1, 10.61 MS (MALDI-TOF) Calcd. for C_106_H_76_F_8_N_4_O_6_: 1653.57 found: [M + H] 1654.48.

## 4. Conclusions

Perylene diimide is as *n*-type organic in this study, four new functional building blocks based on PDI were developed. Styryl and phenol groups have been substituted in the bay region of PDI. For the introduction of these groups in one site of PDI, a novel strategy was developed, utilizing mono nitro PDI in a Suzuki reaction. The PDIs with styryl and phenol groups attached to one site of the perylene skeleton, were easily provided in multi gram scale. Styryl group was used for the substitution of quinoline derivatives via a C-C Heck coupling reaction to afford styryl linked quinoline and PDI molecules. Phenol functionalized PDIs were used in nucleophilic substitution reaction with phenyl perfluorophenyl quinoline to afford ether based quinoline substituted PDIs in higher yields. All new synthesized molecules show intense and broad absorption from 450 to 700 nm. The designed molecules presented good solubility combined with red shifted absorbance and controllable electronic properties. LUMO levels appear to be dictated by the PDI units resulting in low lying LUMOs in the range of −3.8 to −4.2 eV. The functional PDIs could be used as new building blocks in order to develop new small molecules based on PDI.

## Figures and Tables

**Table 1:** Solubility of monosubstituted PDIs.

Solvent	Styryl-di-EH-PDI	PhOH-di-EH-PDI	Ph5FQ-PhO-di-EH-PDI
*o*-DCB	3%	3%	3%
CHCl_3_	3%	3%	3%
DCM	2%	3%	-
THF	2%	3%	-
Toluene	2%	1%	-

**Table 2:** Solubility of disubstituted PDIs.

Solvent	distyryl-di-EH-PDI	diQPy-styryl-di-EH-PDI	diPhOH-di-EH-PDI	diPh5FQ-PhO-di-EH-PDI
*o*-DCB	3%	3%	0.5%	2%
CHCl_3_	3%	3%	0.5%	2%
DCM	3%	2%	0.5%	2%
THF	3%	3%	3%	2%
Toluene	3%	2%	0.5%	2%

**Table 3:** Summary of optical and electrochemical properties of the synthesized PDI-based small molecules.

	λ (nm)			E_opt_^d^ (eV)	E_RED_^e^ (eV)	LUMO (eV)	Homo (eV)
	Solution ^a^	Film ^b^	Onset ^c^				
**distyryl-di-EH-PDI**	556	545	665	1.86	−0.48	−4.12	−5.98
**diPhOH-di-EH-PDI**	570	595	730	1.69	−0.55	−4.05	−5.74
**diQPy-styryl-di-EH-PDI**	365/565	365/565	680	1.82	−0.65	−3.95	−5.77
**diPh5FQ-PhO-di-EH-PDI**	326/558	566	706	1.75	−0.58	−4.02	−5.77
**Styryl-di-EH-PDI**	550	570	726	1.71	−0.43	−4.18	−5.89
**PhOH-di-EH-PDI**	540	535	702	1.77	−0.49	−4.11	−5.88
**Ph5FQ-PhO-di-EH-PDI**	330/535	348/500	690	1.96	−0.80	−3.80	−5.76

^a^ = Absorption maximum in o-DCB. ^b^ = Absorption maximum in film form. ^c^ = onset determined from the onset of the absorbance peak in the longest wavelengths in film form. ^d^ = optical bandgap determined from the equation: $E_{\mathrm{opt}}=\frac{1240}{\lambda_{\mathrm{onset}}}$. ^e^ = determined from the onset of reduction peak from cyclic voltammetry (CV).

## Supplementary Materials

The following are available online at https://www.mdpi.com/1420-3049/24/23/4406/s1. Figure S1. ^1^H NMR spectrum of styryl-di-EH-PDI in CDCl_3_. Figure S2. ^1^H NMR spectrum of PhOH-di-EH-PDI in CDCl_3_. Figure S3. ^1^H NMR spectrum of diPhOH-di-EH-PDI in CDCl_3_. Figure S4. ^1^H NMR spectrum of distyryl-di-EH-PDI in CDCl_3_. Figure S5. ^1^H NMR spectrum of 5FQ-styryl-di-EH-PDI in CDCl_3_. Figure S6. Enlarged aromatic region of ^1^H NMR spectrum of 5FQ-styryl-di-EH-PDI in CDCl_3_. Figure S7. ^1^H NMR spectrum of di5FQ-styryl-di-EH-PDI in CDCl_3._ Figure S8. ^1^H NMR spectrum of diQPy-*styryl*-di-EH-PDI in CDCl_3_. Figure S9. ^1^H NMR spectrum Ph5FQ-PhO-di-EH-PDI in CDCl_3_. Figure S10. ^1^H NMR spectrum of diPh5FQ-PhO-di-EH-PDI in CDCl_3_. Figure S11. ATR-IR spectra of mono substituted perylene diimides and (b) ATR-IR spectra of disubstitued perylene diimides. Figure S12. ^13^C NMR spectrum of styryl-di-EH-PDI in CDCl_3_. Figure S13. ^13^C NMR spectrum of PhOH-di-EH-PDI in CDCl_3_. Figure S14. ^13^C NMR spectrum of distyryl-di-EH-PDI in CDCl_3_. Figure S15. ^13^C NMR spectrum of diQPy-styryl-di-EH-PDI in CDCl_3_. Figure S16. ^13^C NMR spectrum of Ph5FQ-PhO-di-EH-PDI in CDCl_3_. Figure S17. ^13^C NMR spectrum of diPh5FQ-PhO-di-EH-PDI in CDCl_3_. Figure S18. MALDI Spectrum of styryl-di-EH-PDI. Figure S19. MALDI Spectrum of diPhOH-di-EH-PDI. Figure S20. MALDI Spectrum of diQPy-styryl-di-EH-PDI. Figure S21. MALDI Spectrum of Ph5FQ-PhO-di-EH-PDI. Figure S22. MALDI spectrum of diPh5FQ-PhO-di-EH-PDI.
