# Supplementary material for: Synthesis and Optoelectronic Characterization of Perylene Diimide-Quinoline Based Small Molecules
Source: Molecules. 2019 Dec 2;24(23):4406. doi: 10.3390/molecules24234406 (PMC6930630; doi:10.3390/molecules24234406)
Supplement: Supplementary file 1 [file molecules-24-04406-s001.pdf]

Supporting Information

Synthesis and optoelectronic characterization of Perylene Diimide – Quinoline based small molecules

Stefania Aivali[1], Loukia. Tsimpouki[1], C. Anastasopoulos[1,2], J. K. Kallitsis[1,2*]

[1] Department of Chemistry, University of Patras, University Campus, Rio-Patras, GR26504, Greece; j.kallitsis@upatras.gr
[2] Foundation for Research and Technology Hellas/Institute of Chemical Engineering Sciences (FORTH/ICE-HT), Platani Str., Patras, GR26504, Greece; j.kallitsis@upatras.gr
* Correspondence: j.kallitsis@upatras.gr;

^{1}H, ^{13}C NMR and ATR-IR spectra of the synthesized molecules

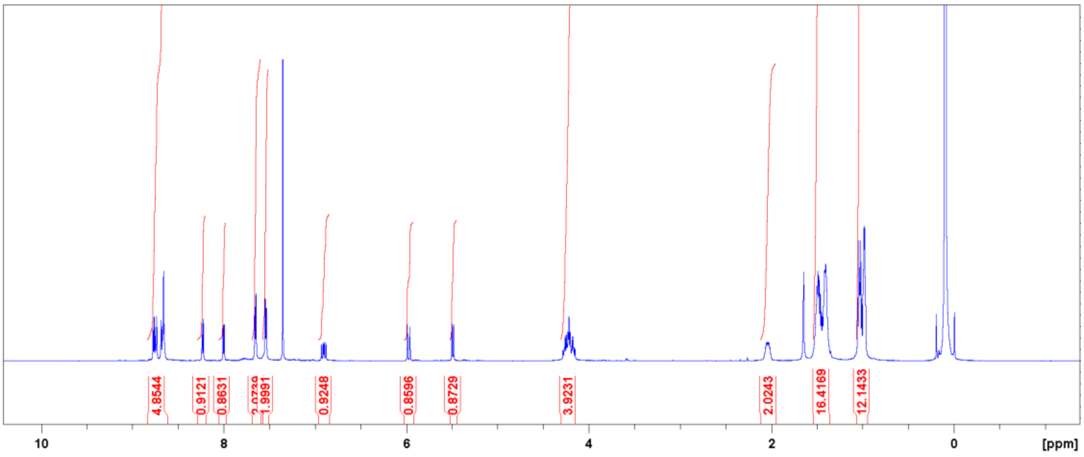

Figure S1. ^{1}H NMR spectrum of styryl-di-EH-PDI in $CDCl_3$.

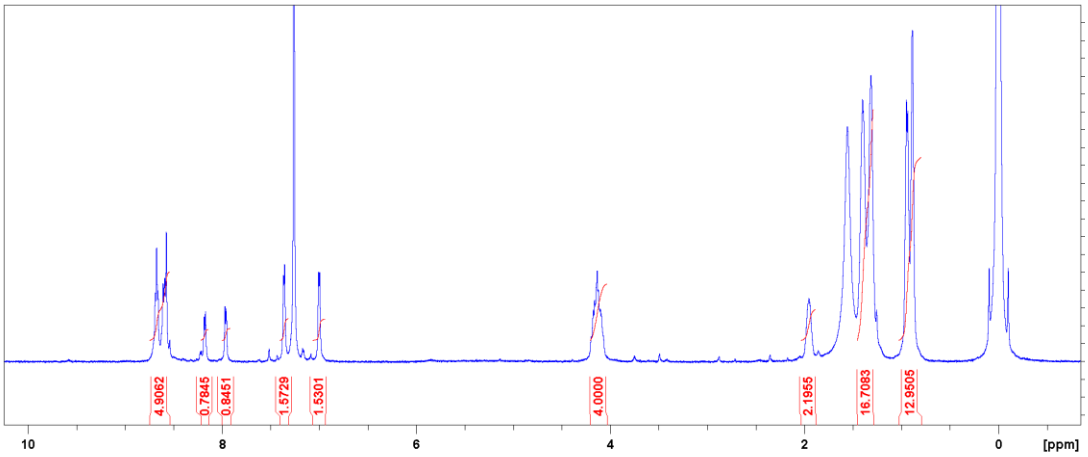

Figure S2. ^{1}H NMR spectrum of PhOH-di-EH-PDI in $CDCl_3$.

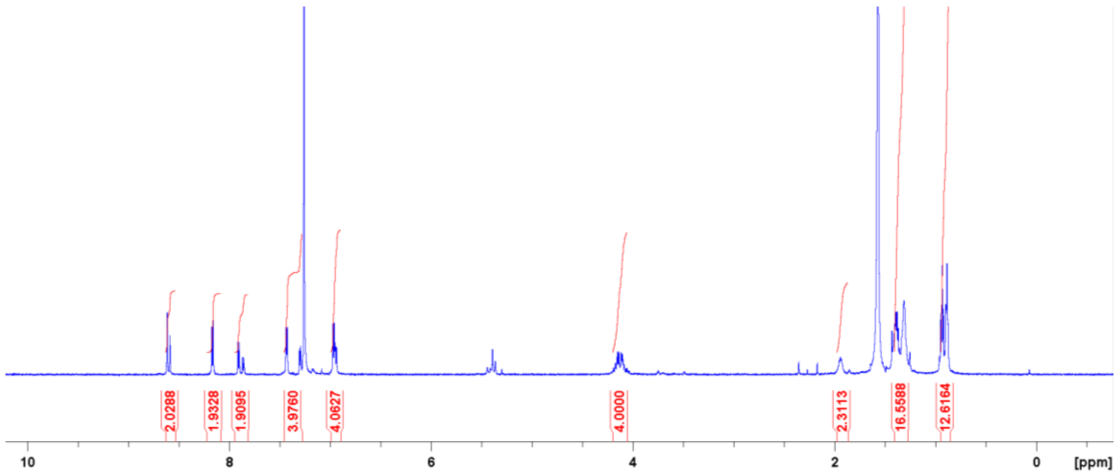

Figure S3. ^{1}H NMR spectrum of diPhOH-di-EH-PDI in $CDCl_3$.

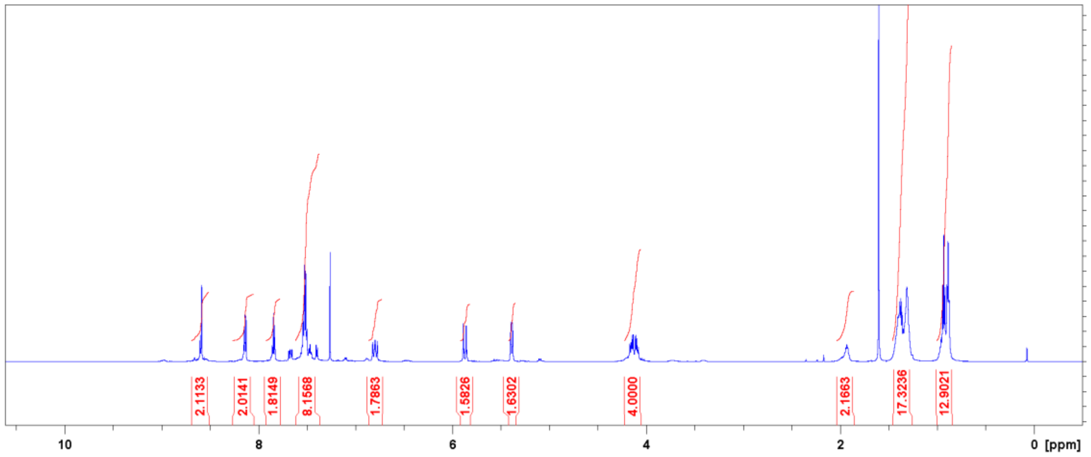

Figure S4. ^{1}H NMR spectrum of distyryl-di-EH-PDI in $CDCl_3$.

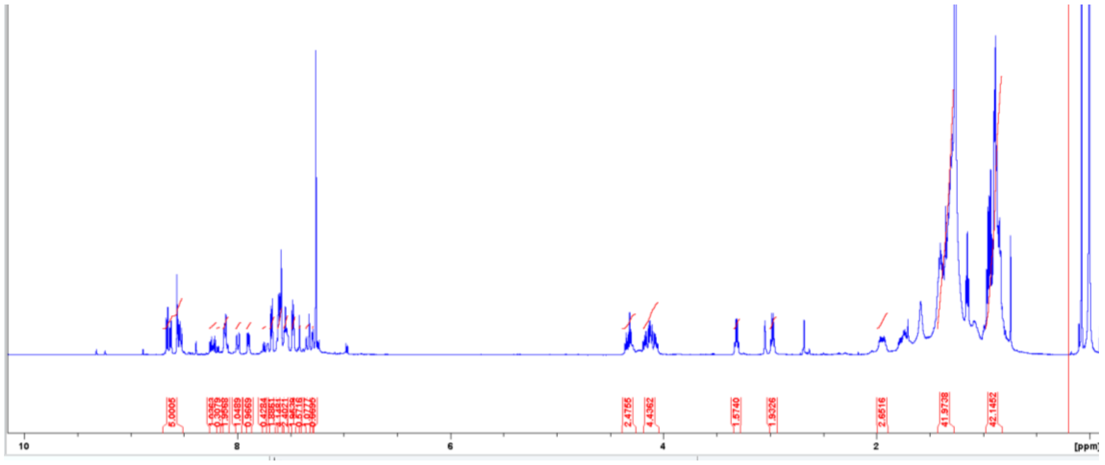

Figure S5. ^{1}H NMR spectrum of 5FQ-styryl-di-EH-PDI in $CDCl_3$.

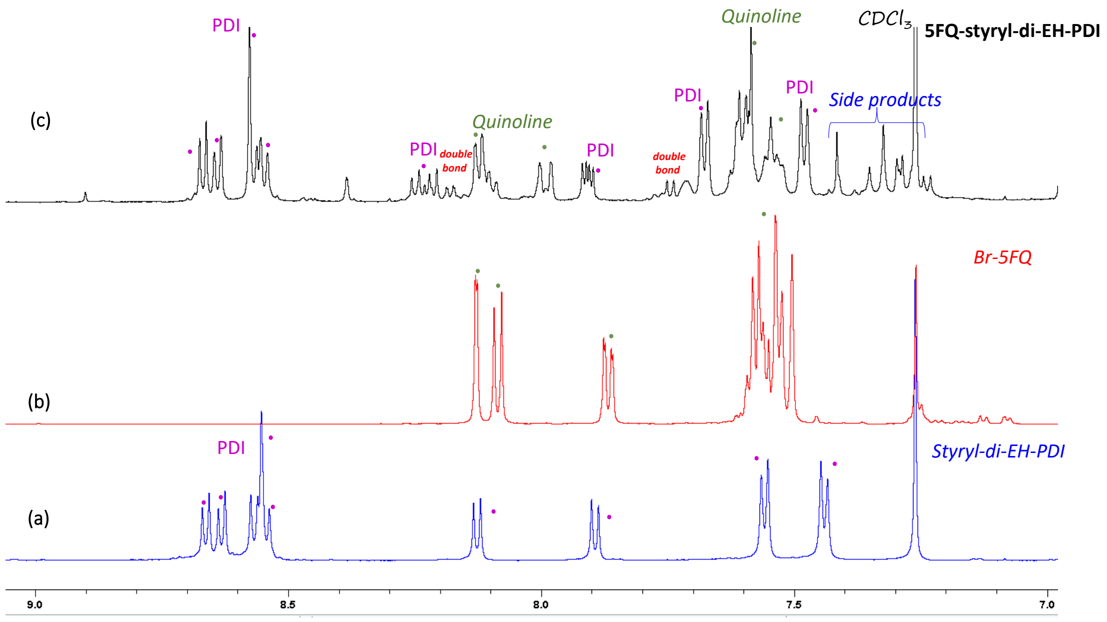

Figure S6: Enlarged aromatic region of ^{1}H NMR spectrum of 5FQ-styryl-di-EH-PDI in $CDCl_3$.

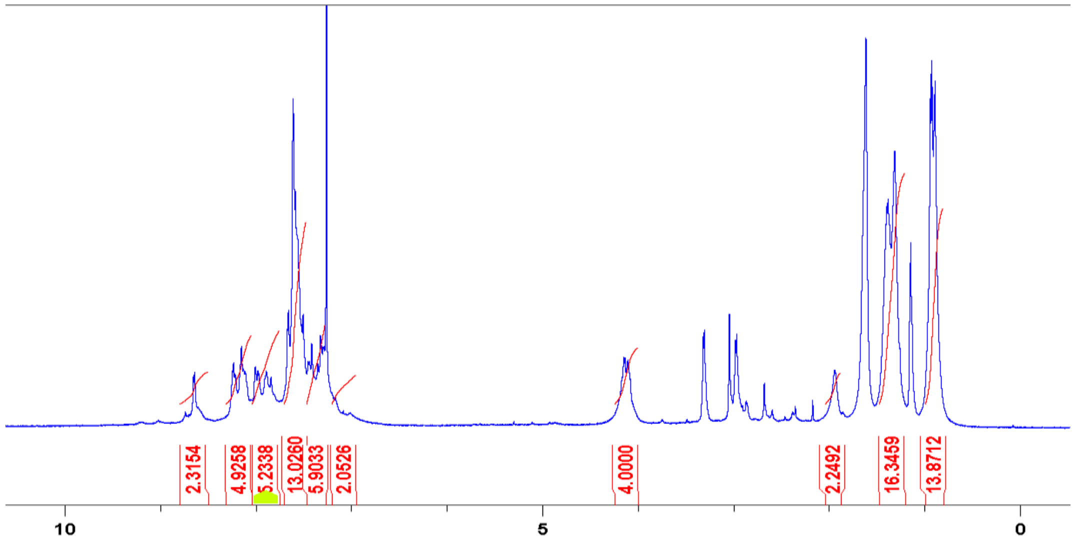

Figure S7. ^{1}H NMR spectrum of di5FQ-styryl-di-EH-PDI in $CDCl_3$.

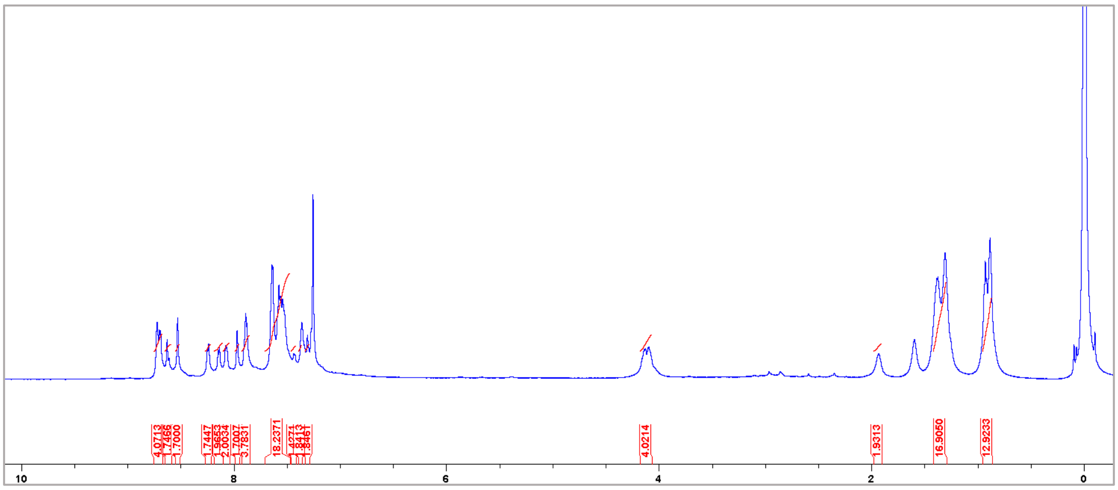

Figure S8. ^{1}H NMR spectrum of diQPy-styryl-di-EH-PDI in $CDCl_3$.

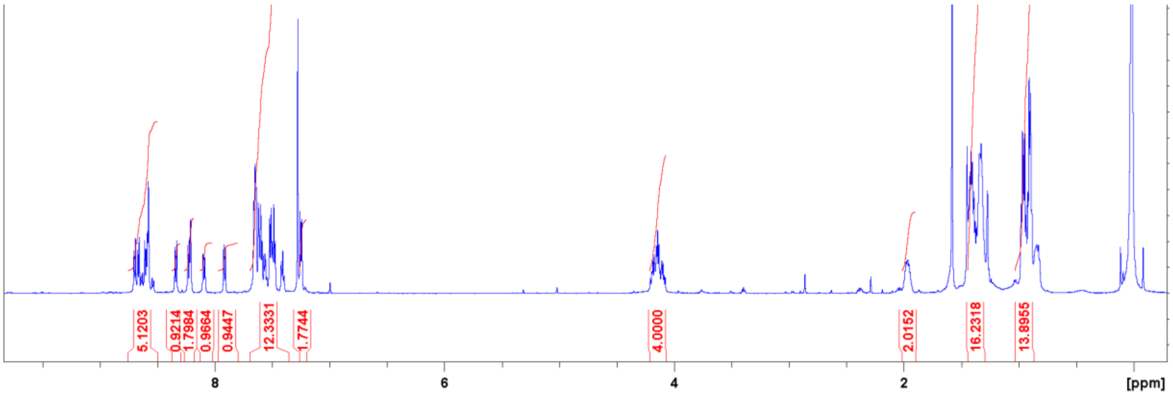

Figure S9. ^{1}H NMR spectrum of Ph5FQ-PhO-di-EH-PDI in $CDCl_3$.

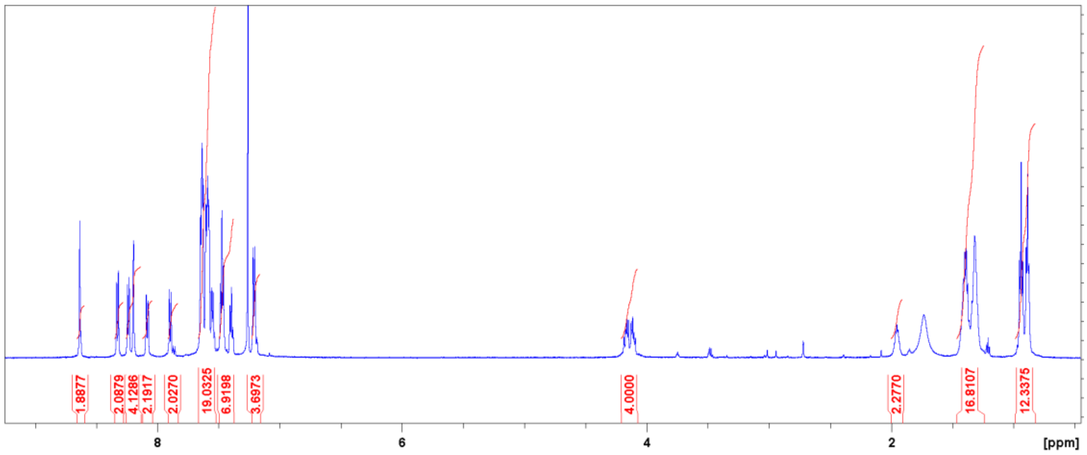

Figure S10.^{1}H NMR spectrum of diPh5FQ-PhO-di-EH-PDI in $CDCl_3$.

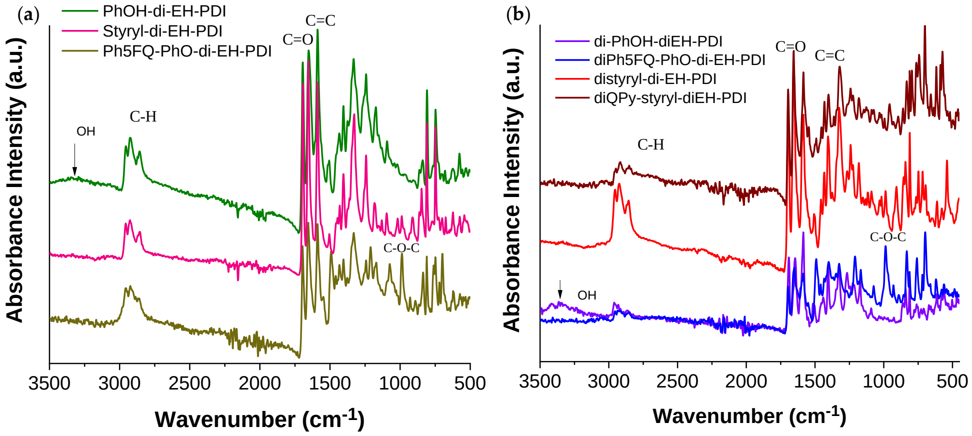

Figure S11 .ATR-IR spectra of mono substituted perylene diimides and (b) ATR-IR spectra of disubstituted perylene diimides.

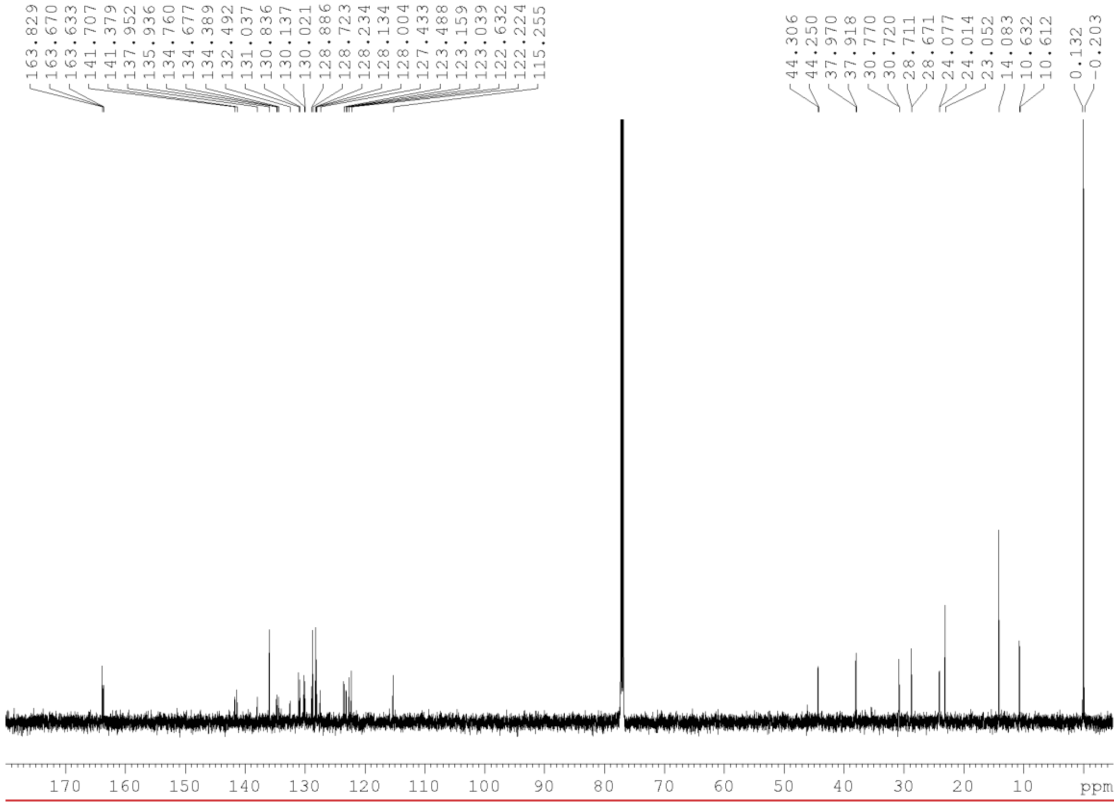

Figure S12. ^{13}C NMR spectrum of styryl-di-EH-PDI in $CDCl_3$.

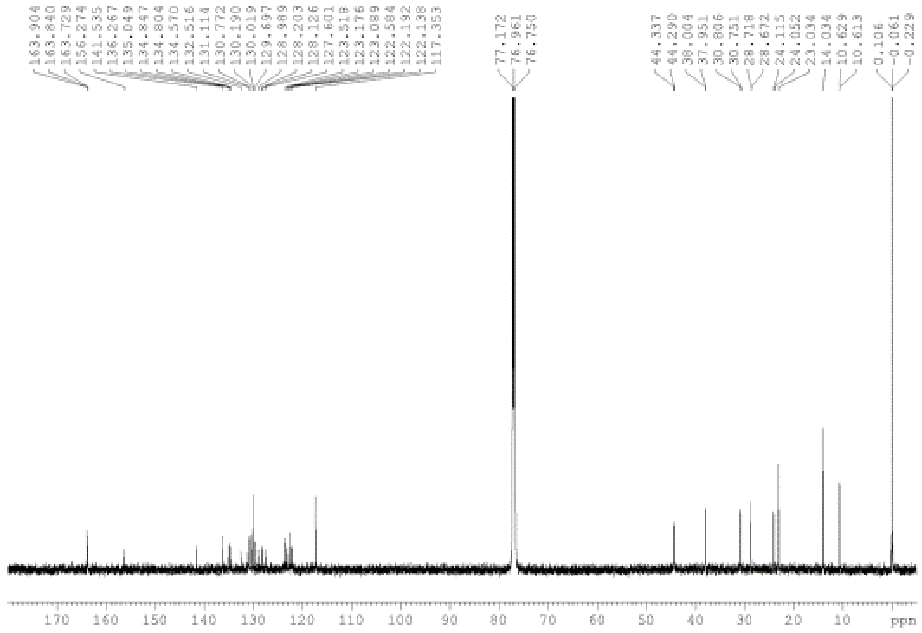

Figure S13. ^{13}C NMR spectrum of PhOH-di-EH-PDI in $CDCl_3$.

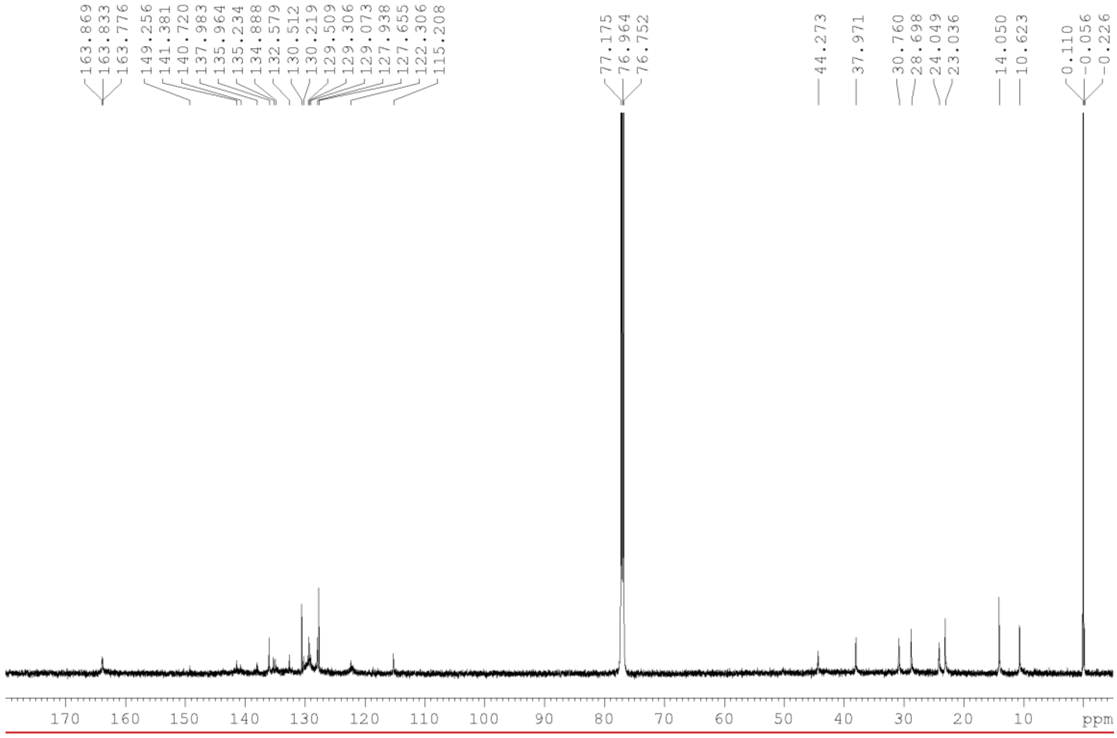

Figure S14. ^{13}C NMR spectrum of distyryl-di-EH-PDI in $CDCl_3$.

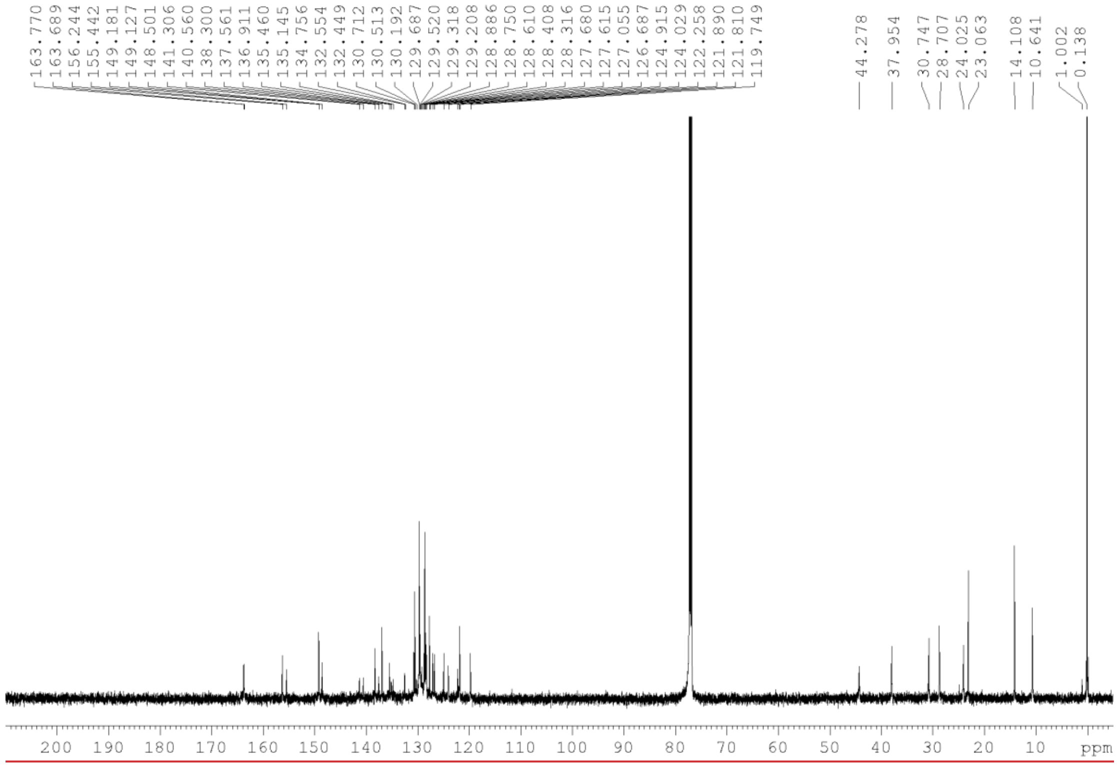

Figure S15. ^{13}C NMR spectrum of diQPy-styryl-di-EH-PDI in $CDCl_3$.

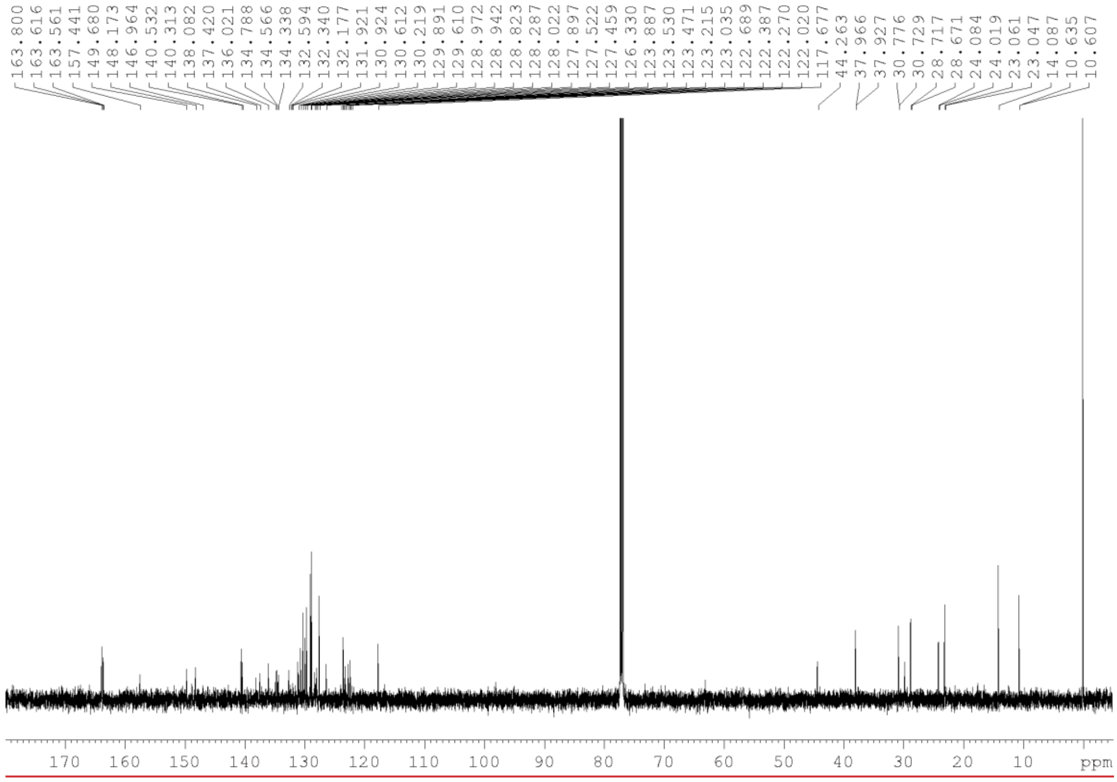

Figure S16. ^{13}C NMR spectrum of Ph5FQ-PhO-di-EH-PDI in $CDCl_3$.

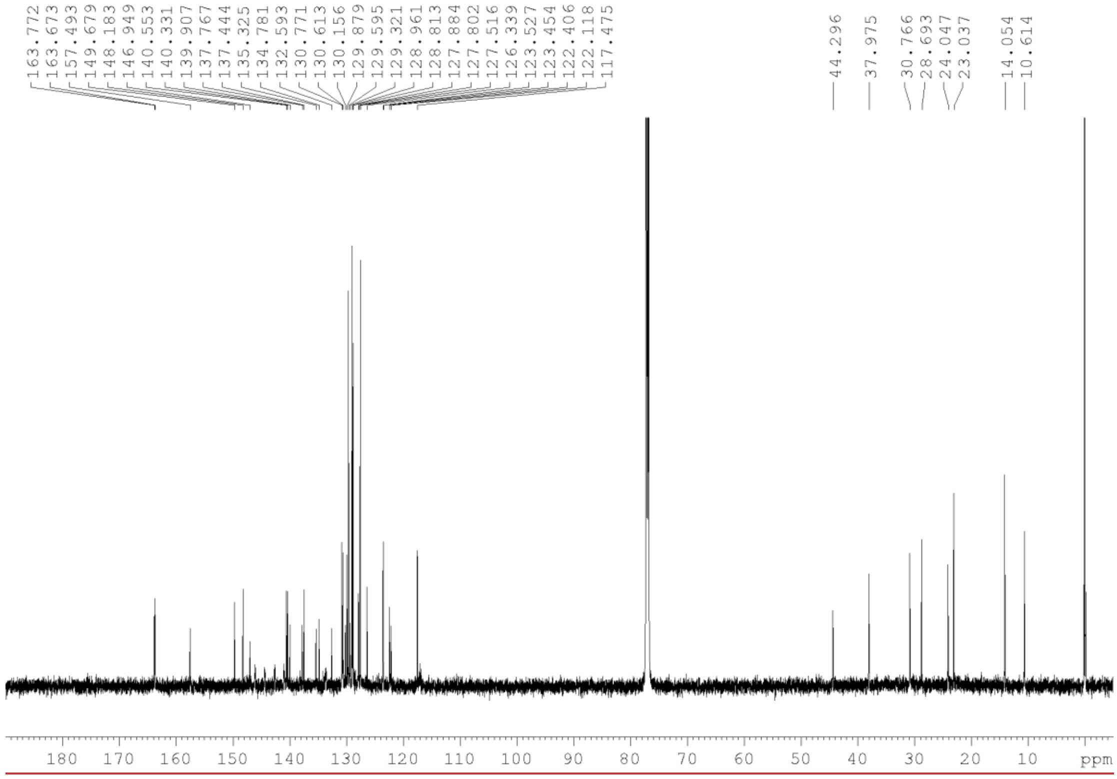

Figure S17. ^{13}C NMR spectrum of diPh5FQ-PhO-di-EH-PDI in $CDCl_3$.

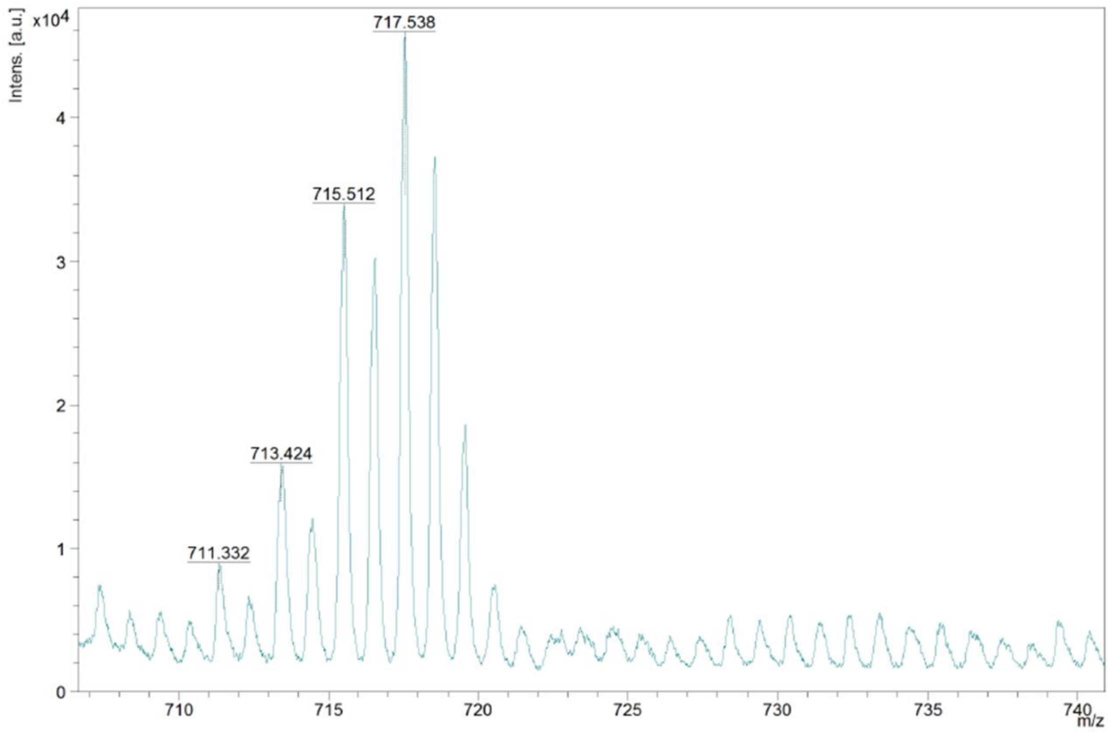

Figure S18 MALDI Spectrum of styryl-di-EH-PDI.

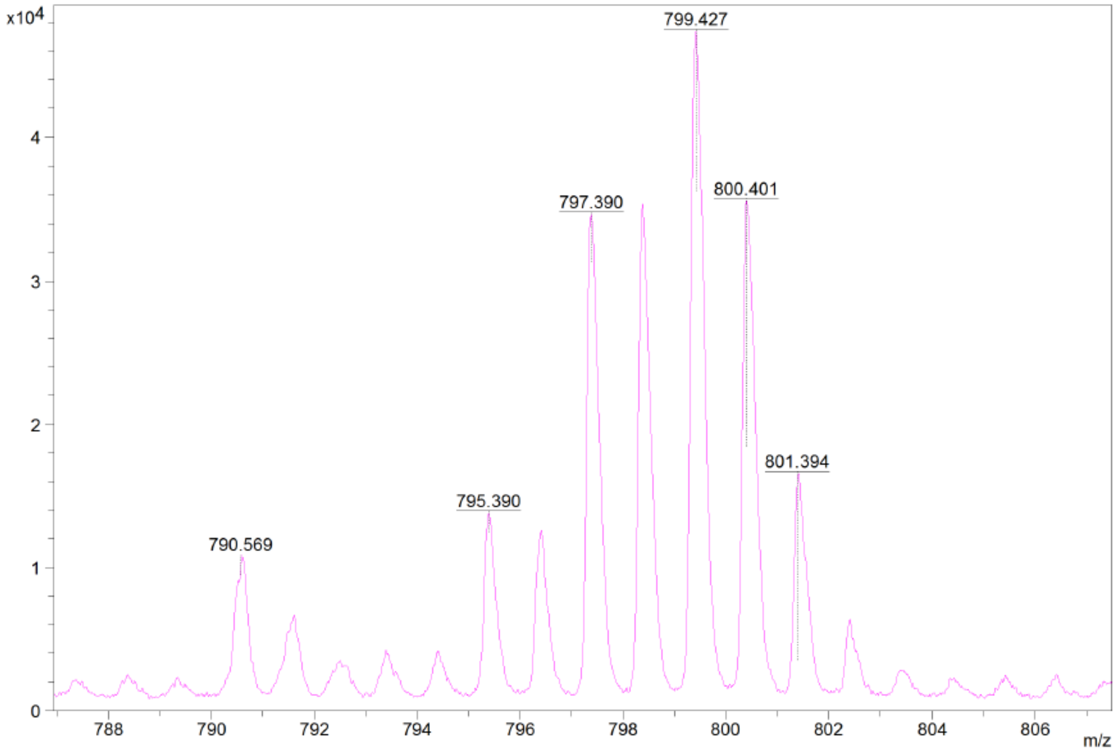

Figure S19. MALDI Spectrum of diPhOH-di-EH-PDI.

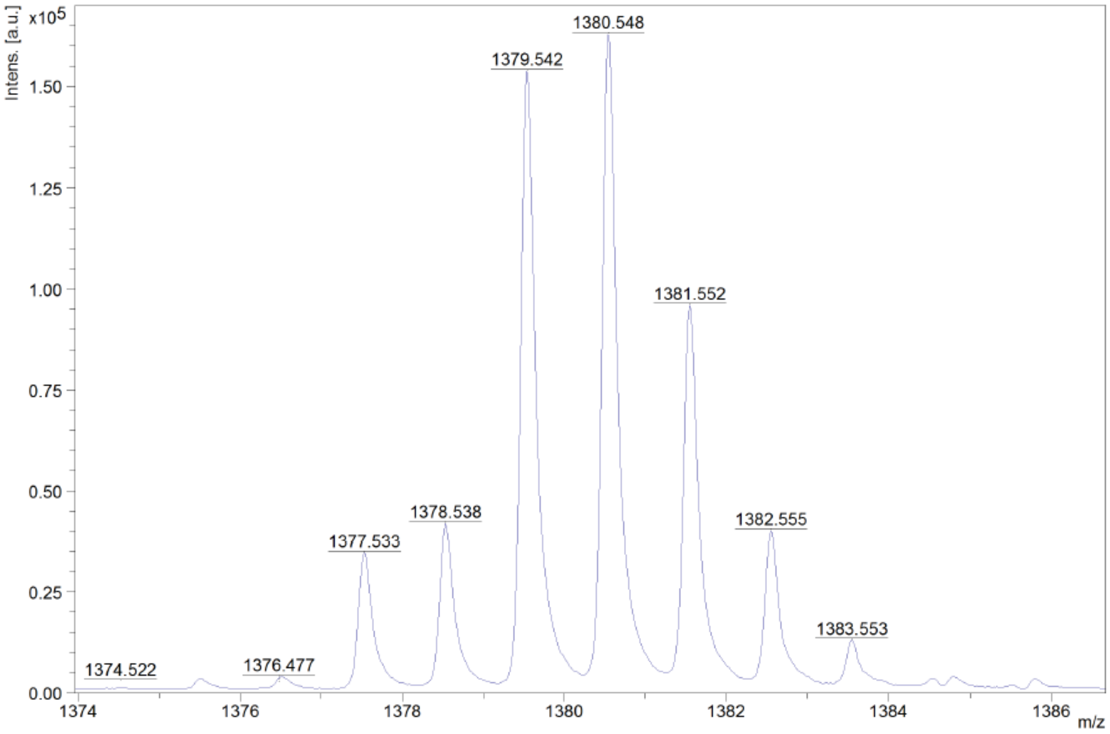

Figure S20. MALDI Spectrum of diQPy-styryl-di-EH-PDI.

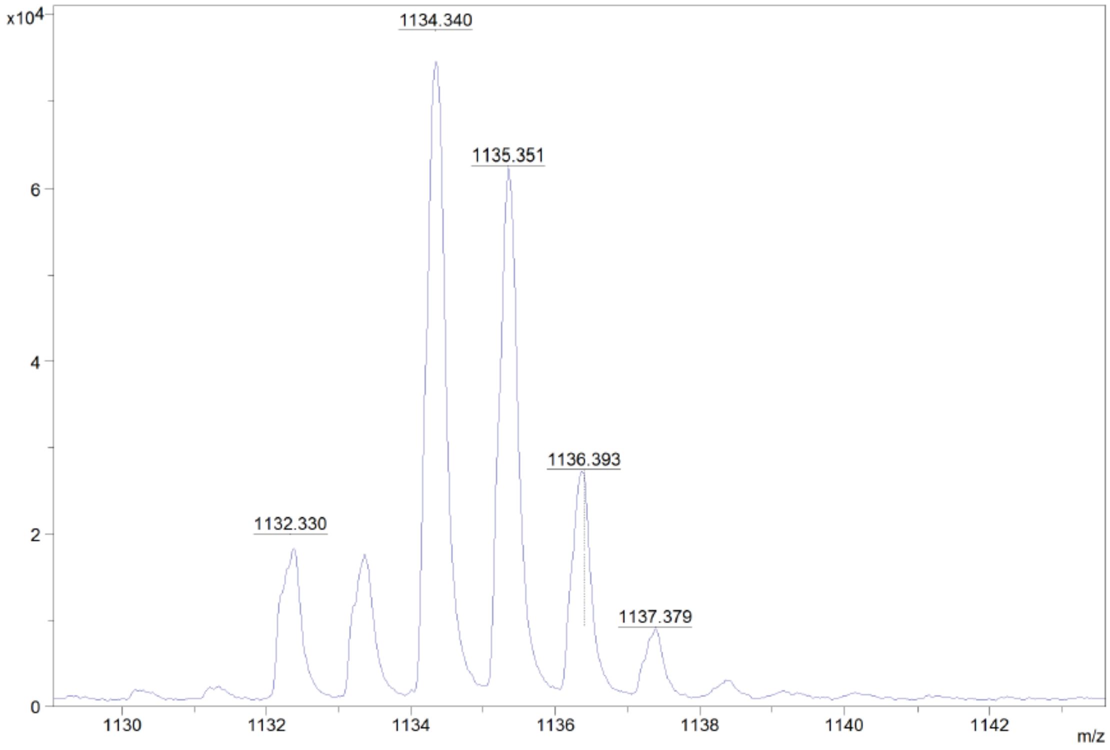

Figure S21. MALDI Spectrum of Ph5FQ-PhO-di-EH-PDI.

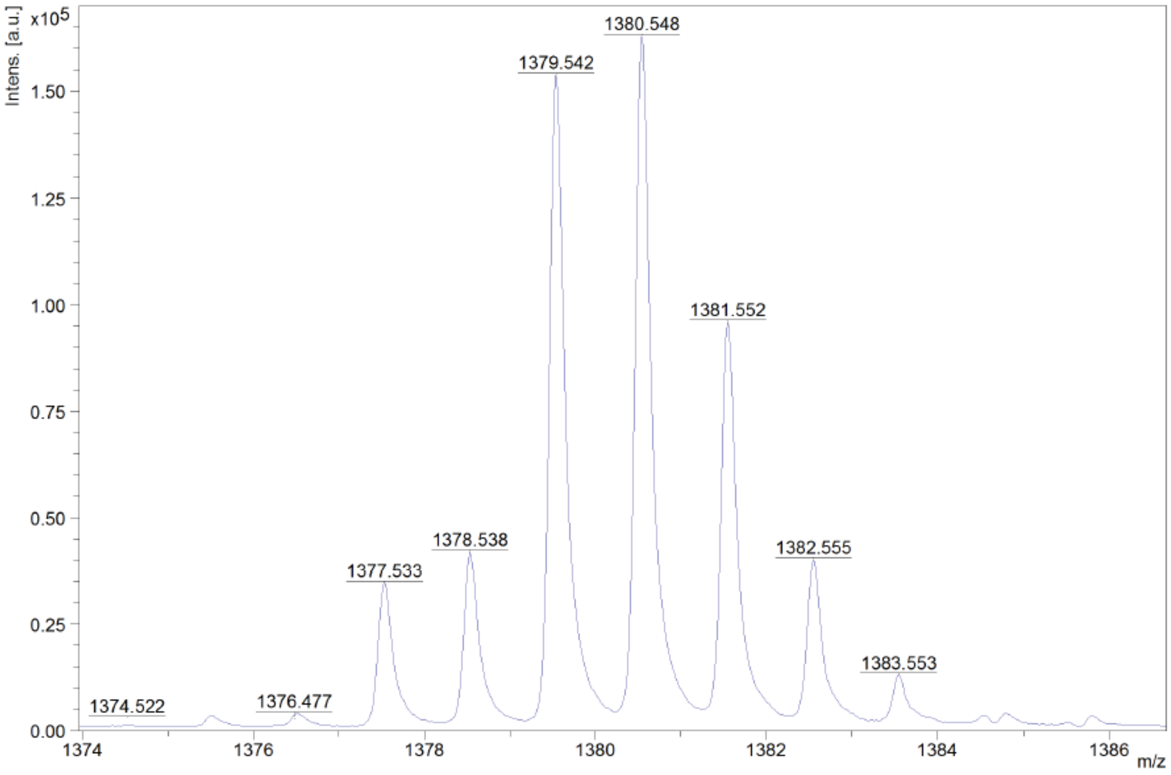

Figure S22. MALDI spectrum of diPh5FQ-PhO-di-EH-PDI.
